# Prevalence and clinical relevance of helminth co-infections among tuberculosis patients in urban Tanzania

**DOI:** 10.1371/journal.pntd.0005342

**Published:** 2017-02-08

**Authors:** Francis Mhimbira, Jerry Hella, Khadija Said, Lujeko Kamwela, Mohamed Sasamalo, Thomas Maroa, Magreth Chiryamkubi, Grace Mhalu, Christian Schindler, Klaus Reither, Stefanie Knopp, Jürg Utzinger, Sébastien Gagneux, Lukas Fenner

**Affiliations:** 1 Department of Intervention and Clinical Trials, Ifakara Health Institute, Dar es Salaam, Tanzania; 2 Department of Medical Parasitology and Infection Biology, Swiss Tropical and Public Health Institute, Basel, Switzerland; 3 University of Basel, Basel, Switzerland; 4 Department of Curative Services, Ministry of Health, Community Development, Gender, Elderly and Children, Dar es Salaam, Tanzania; 5 Department of Epidemiology and Public Health, Swiss Tropical and Public Health Institute, Basel, Switzerland; 6 Wolfson Wellcome Biomedical Laboratories, Department of Life Sciences, Natural History Museum, London, United Kingdom; 7 Institute of Social and Preventive Medicine, University of Bern, Bern, Switzerland; George Washington University, UNITED STATES

## Abstract

**Background:**

Helminth infections can negatively affect the immunologic host control, which may increase the risk of progression from latent *Mycobacterium tuberculosis* infection to tuberculosis (TB) disease and alter the clinical presentation of TB. We assessed the prevalence and determined the clinical relevance of helminth co-infection among TB patients and household contact controls in urban Tanzania.

**Methodology:**

Between November 2013 and October 2015, we enrolled adult (≥18 years) sputum smear-positive TB patients and household contact controls without TB during an ongoing TB cohort study in Dar es Salaam, Tanzania. We used Baermann, FLOTAC, Kato-Katz, point-of-care circulating cathodic antigen, and urine filtration to diagnose helminth infections. Multivariable logistic regression models with and without random effects for households were used to assess for associations between helminth infection and TB.

**Principal findings:**

A total of 597 TB patients and 375 household contact controls were included. The median age was 33 years and 60.2% (585/972) were men. The prevalence of any helminth infection among TB patients was 31.8% (190/597) and 25.9% (97/375) among controls. *Strongyloides stercoralis* was the predominant helminth species (16.6%, 161), followed by hookworm (9.0%, 87) and *Schistosoma mansoni* (5.7%, 55). An infection with any helminth was not associated with TB (adjusted odds ratio (aOR) 1.26, 95% confidence interval (CI): 0.88–1.80, p = 0.22), but *S*. *mansoni* infection was (aOR 2.15, 95% CI: 1.03–4.45, p = 0.040). Moreover, *S*. *mansoni* infection was associated with lower sputum bacterial load (aOR 2.63, 95% CI: 1.38–5.26, p = 0.004) and tended to have fewer lung cavitations (aOR 0.41, 95% CI: 0.12–1.16, p = 0.088).

**Conclusions/Significance:**

*S*. *mansoni* infection was an independent risk factor for active TB and altered the clinical presentation in TB patients. These findings suggest a role for schistosomiasis in modulating the pathogenesis of human TB. Treatment of helminths should be considered in clinical management of TB and TB control programs.

## Author summary

Tuberculosis (TB), caused by the bacterium *Mycobacterium tuberculosis*, and parasitic worm infections are typical diseases of poverty. They often overlap geographically, and can occur in the same individual. Parasitic worm infections contribute to the down-regulation of the essential immune response against TB, and therefore can increase progression from latent *M*. *tuberculosis* infection to active TB. We conducted a case-control study in Dar es Salaam, the economic capital of Tanzania, where TB and helminths constitute a considerable burden. We found that infection with the blood fluke *Schistosoma mansoni* was associated with active TB, while none of the other parasitic worms showed such an association. Interestingly, TB patients infected with *S*. *mansoni* had significantly lower sputum bacterial load at diagnosis and tended to have fewer lung cavitations compared with TB patients without any parasitic worm infection. Diagnosis and treatment of parasitic worm infections, particularly schistosomiasis, should be considered during the management of TB patients and in the context of TB control programs. This could help to reduce the TB burden in settings where TB and parasitic worms co-exist.

## Introduction

Tuberculosis (TB), caused by *Mycobacterium tuberculosis* remains a challenging disease to control. Indeed, over two billion people are estimated to be infected with *M*. *tuberculosis* worldwide [1]. Moreover one billion people are infected with soil-transmitted helminths, schistosomes, filarial worms, and food-borne trematodes [2–4]. In 2014, an estimated 9.6 million new TB patients were notified and 1.5 million TB patients died from the disease [1]. TB is a leading cause of deaths from an infectious disease [5].

TB and helminthiases overlap geographically, particularly in areas where poverty persists, for example in countries of sub-Saharan Africa [1,6]. Where TB and helminth infections co-occur, they can affect the same individual and thus exacerbate the course of disease [6]. Several conditions such as diabetes mellitus, malnutrition, and malignancies are known to increase the risk of progressing from latent *M*. *tuberculosis* infection to active TB [7]. Human immunodeficiency Virus (HIV)-induced immunodeficiency is by far the most important risk factor for developing TB [1,8], but parasitic co-infections such as with helminths can also contribute to the development of TB [9–11]. Immune dysregulations caused by helminth infections are known to negatively affect the prognosis of HIV and malaria [6,12]. The immune response to helminth infections is characterized by the induction of CD4^+^ T-helper 2 (Th2) and down-regulation of CD4^+^ T-helper 1 (Th1) cells [12–15]. This immunological imbalance has been suggested to increase the risk of progression from latent *M*. *tuberculosis* infection to active TB and to worsen the clinical outcomes.

We aimed to study the interaction between TB and helminth co-infections by comparing the prevalence of helminth infections, using a suite of diagnostic techniques, between TB patients and household contact controls without TB in an ongoing cohort study in Dar es Salaam, Tanzania, and to assess the effects of helminth infection on the clinical presentation and outcomes of TB disease.

## Methods

### Ethics statement

The study protocol was approved by the institutional review board of the Ifakara Health Institute (IHI; reference no. IHI/IRB/No 04–2015) and the Medical Research Coordinating Committee of the National Institute of Medical Research (NIMR; reference no. NIMR/HQ/R.8c/Vol.I/357) in Tanzania, and the ethics committee of north-west and central Switzerland (EKNZ; reference no.: UBE-15/42). Written informed consent was obtained from all study participants. TB patients were treated according to the National TB and Leprosy Programme (NTLP) treatment guideline [8]. Individuals with a *Schistosoma* spp. infection were treated with praziquantel (40 mg/kg). Other helminth infections were treated with albendazole (400 mg) immediately after diagnosis, as recommended by the national treatment guidelines [16]. HIV-positive patients were clinically managed according to the Tanzania National HIV and acquired immune deficiency syndrome (AIDS) treatment guideline [17].

### Study setting

The study was conducted in the densely populated urban setting of Temeke district in Dar es Salaam, which is the economic capital of Tanzania. The population of Temeke is estimated at 1.4 million. In 2014, about one third of all TB patients from Dar es Salaam were notified in Temeke district (4,373; 32%) [18]. The overall HIV prevalence in the general adult population in Dar es Salaam is 5.2% [19]. The study area includes two TB sub-districts, Wailes I and Wailes II, whose patients are clinically managed at the Temeke district hospital and the two associated TB diagnostic and treatment centers of Tambukareli and Pasada [20].

### Study design

The study was conducted within the frame of an ongoing prospective cohort study of TB patients and household contact controls in Dar es Salaam (TB-DAR). We assessed the association of TB and helminth infection in a case-control study design of TB patients (sputum smear-positives for acid-fast bacilli [AFB]) and household contact controls (Xpert MTB/RIF negative), who were matched by age (±5 years) and whenever possible by sex. We prospectively followed-up TB patients and assessed the clinical outcomes comparing TB patients with and without helminth infection at 6 and 12 months after recruitment.

### Study population and sample size

We consecutively enrolled study participants starting in November 2013 until October 2015 to reach the required sample size. Over this period, we included adult TB patients (≥18 years of age and sputum-smear positive) and household contact controls. Any individual living in the same household as the index TB patients enrolled in the study is referred to as a household contact control. Controls at recruitment were free of symptoms and signs suggestive of TB, healthy on physical examination, and had a negative Xpert MTB/RIF result (Cepheid; California, United States of America).

Assuming a helminth prevalence of 45% in TB patients and 26% in controls based on results from previous publications [21] and a power of 80%, the target sample size was 109 study participants (for each group) to detect a prevalence difference of 19% between the two groups with a significance level of test 0.05, two-tailed and calculated with Stata version 14.0 (Stata Corp; Texas, United States of America).

### Study procedures

TB patients and household contact controls were interviewed and underwent physical examination during recruitment at the study site (see under “Data Collection and Definitions”). We collected skinfold measurements from four body sites (biceps, triceps, subscapular, and suprailiac) using the Harpenden skinfold caliper [22]. The percentage body fat was calculated as previously described [23]. Household contacts with no symptoms or signs of TB submitted a sputum sample for Gene Xpert MTB/RIF to rule-out TB. We collected blood, stool, and urine samples from TB patients and controls for subsequent laboratory investigations. Chest X-rays for TB patients were done at the Temeke district hospital and were interpreted by an experienced board certified radiologist who was blinded to patients’ clinical data. Trained field workers collected geographic coordinates (global positioning system [GPS]) from the patients’ homes using Samsung Tab 4 android tablets (Samsung; Suwon, South Korea).

### Laboratory procedures

#### Microbiological investigations

A patient was considered as having TB when any of the two submitted sputum samples were positive for AFB by staining sputum smears using the Ziehl-Nielsen (ZN) method, and a positive mycobacterial culture. Sputum smear microscopy was done at the Temeke district hospital under continuous quality control by the central tuberculosis reference laboratory (Dar es Salaam, Tanzania). AFB smear-positive results were graded according to World Health Organization/International Union Against Tuberculosis and Lung Disease (WHO/IUATLD) guidelines: “scanty” with 1–9 AFB per 100 oil immersion fields; “1+” with 10–99 AFB per 100 immersion fields; “2+” with 1–10 AFB per 1 immersion field, and “3+” with >10 AFB per immersion field [8,24]. To rule out TB among household controls, an additional sputum sample from TB patients and controls was sent to the TB laboratory at the Bagamoyo Research and Training Center (BRTC), IHI, for GeneXpert MTB/RIF (controls) and for culture on Löwenstein-Jensen media (TB patients and controls).

#### Helminthological investigations

For the diagnosis of helminth infections, single stool and urine samples were collected from each participant before the start of TB treatment (TB patients) and at the time of enrolment (controls). All stool and urine samples were transferred to the Helminth Unit at BRTC and examined for helminth infections using standardized, quality-controlled procedures as described elsewhere [25–27]. The Kato-Katz (triplicate thick smears per stool sample) and the FLOTAC methods were used to diagnose *Ascaris lumbricoides*, hookworm, *S*. *mansoni*, and *Trichuris trichiura* infections. The Baermann method was used to identify *Strongyloides stercoralis* infections [28]. The adhesive tape test was used to diagnose Enterobius vermicularis infections [26]. In addition, a rapid point-of-care circulating cathodic antigen (POC-CCA) urine cassette test was employed for the diagnosis of *S*. *mansoni* [29]. The urine filtration method was applied to detect *S*. *haematobium* infections [26]. For quality control, 10% of Kato-Katz slides were randomly selected and re-examined by a second reader.

#### Blood testing

In line with national HIV testing algorithms, screening was done using the Alere Determine HIV rapid test (Alere, USA). The Uni-gold HIV (Trinity Biotech; Wicklow, Ireland) rapid test served as a confirmatory test in case of a positive screening test. The CD4^+^ T-cells counts were determined using a FACSCount machine (Becton Dickinson Biosciences; California, United States of America). A full blood cell count was done with a MS4 Vet hematology analyzer (Diamond Diagnostics; Massachusetts, United States of America). All blood tests were performed at the Temeke district hospital laboratory, which is under supervision and quality control by the regional laboratory technician.

### Data collection and definitions

We collected socio-demographic indicators including age, sex, ethnicity, education, and household income. Anthropometric data included weight, height, and skinfold measurements. Clinical data collected pertained to presenting symptoms of TB patients, TB treatment category, and treatment outcomes. Laboratory data included ZN sputum smear results and Gene Xpert MTB/RIF results, helminth species infections, HIV status, full blood cell count, and CD4^+^ cell count. All study participants were asked about their use of anthelmintic treatment in the last 12 months prior to the enrollment into the study. Study data were captured by electronic case report forms using the open-source data collection software ODK on Android PC tablets [30]. Data management was done using the *e*Management tool “odk_planner”, as previously described [30]. Data were uploaded to a password protected secure server with regular back-ups.

In order to grade the clinical severity of TB, we adopted a previously published clinical TB score [31], with the following modification: 12 points TB score parameters instead of 13 points as tachycardia was not systematically measured. The following TB score parameters were used: (i) coughing; (ii) hemoptysis; (iii) chest pain; (iv) dyspnea; (v) night sweating; (vi) anemic conjunctivae; (vii) positive finding at auscultation; (viii) axillary temperature >37.0°C; (ix) mid upper arm circumference (MUAC) <220 mm; (x) MUAC <200 mm; (xi) body mass index (BMI) <18 kg/m^2^; and (xii) BMI <16 kg/m^2^. TB score was then categorized into mild (score of 1–5) and severe (score of ≥6). Low BMI was defined as BMI <18 kg/m^2^; high sputum bacterial load as AFB sputum smear result ≥2+ (quantitative scoring), which correlates with GeneXpert Ct values [32]. To assess the clinical outcomes among TB patients, we defined poor gain as a change in absolute body weight (<7 and ≥7 kg), BMI (<2.6 and ≥2.6 kg/m^2^) and body fat (<0 and ≥0%) from recruitment to month 6 of follow-up.

“Any helminth infection” was defined as infection with any of the following helminth species: *A*. *lumbricoides*, *E. vermicularis*, hookworm, *Hymenolepis diminuta*, *S*. *haematobium*, *S*. *mansoni*, *S*. *stercoralis* and *T*. *trichiura*. High occupational risk for schistosomiasis was defined as working in rice fields, sand harvesting, washing cars, and fishing in freshwater. The intensity of helminth infection was defined according to WHO classification [33]. The average egg counts from the triplicate Kato-Katz thick smears per stool sample and per individual were multiplied by a factor of 24 to obtain eggs per gram (EPG) of stool [25].

### Statistical analysis

We compared the characteristics of TB patients and household contact controls at the time of TB diagnosis or enrolment. The prevalence of helminth infection was calculated from the generalized estimations equation adjusting for clustering at the household level. We used multilevel mixed-effects logistic regression with random intercepts at the level of households to assess risk factors for helminth infection. To assess risk factors for TB, we compared cases and controls using unconditional logistic regression because not all TB cases could be assigned a control. In addition, we also performed conditional logistic regression among matched pairs to confirm the results. Additional analyses assessed the association of TB and with specific helminth species separately. We also examined whether the association between the presence of a helminth infection and a recent history of deworming drugs depended on HIV infection status by including an interaction term in the logistic regression model. Among TB patients, logistic regression models were used to study associations between helminth infection and clinical presentation at the time of TB diagnosis (such as TB score, high sputum bacterial load, lung infiltration, and cavitation), and to study the association between helminth infection and clinical outcomes after 6 months of TB treatment (change in absolute weight, BMI, and percentage body fat). Associations were expressed as crude odds ratios (ORs) and adjusted ORs (aORs). All analyses were performed in Stata version 14.0 (Stata Corp; Texas, United States of America).

We used the geographic coordinates of the TB patients’ homes to analyze the spatial distribution of TB and helminth co-infections. The prevalence of helminths and helminth species was analyzed at the ward level for optimal readability. The average area per ward in the Dar es Salaam region is 15.5 km^2^ [19]. The maps were produced using the software package ArcGIS Desktop version 10.2 (ESRI; California, United States of America) and the shape files from the National Bureau of Statistics of Tanzania [34].

## Results

### Characteristics of study participants

**Fig 1:**
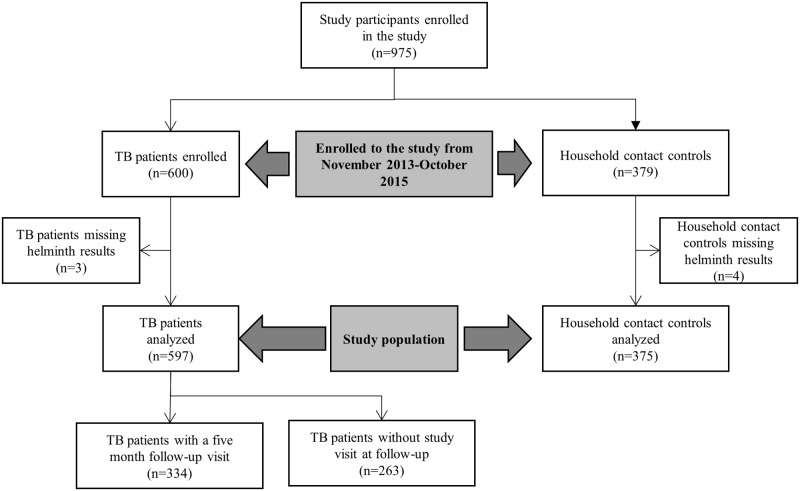
Study participants’ flow diagram.

A total of 597 TB patients and 375 household contact controls were included. Table 1 summarizes the socio-demographic and clinical characteristics of TB patients and controls. The study participants’ flow diagram is shown in Fig 1. Among all study participants, the median age was 33 years (interquartile range [IQR]: 26–41 years) and 60.2% (585/972) were men. HIV prevalence was 20.4% (95% confidence interval (CI): 17.9–23.0%). TB patients were more frequently male compared with controls (68.8% [411/597] *vs*. 46.4% [174/375]), HIV-positive (27.3% [163] *vs*. 9.3% [35]), and smokers (18.1% [108] *vs*. 8.8% [33]). TB patients also had a lower median BMI (18.3 kg/m^2^, IQR: 16.5–20.4 kg/m^2^ *vs*. 23.9 kg/m^2^, IQR: 21.6–28.1 kg/m^2^) and a lower median hemoglobin level (11.3 g/dl, IQR: 9.9–12.7 g/dl *vs*. 12.8 g/dl, IQR: 11.5–14.1 g/dl). The patient characteristics, stratified by HIV status, are shown in S1 Table.

**Table 1:** Socio-demographic and clinical characteristics of tuberculosis (TB) patients and household contact controls without TB.

Characteristics	Total (n = 972)	TB patient (n = 597)	Controls (n = 375)
**Age in years, median (IQR)**	33 (26–41)	33 (26–40)	33 (26–42)
**Age groups (years)**			
18–24	194 (20.0)	107 (17.9)	87 (23.2)
25–34	347 (35.7)	226 (37.9)	121 (32.3)
35–44	266 (27.4)	169 (28.3)	97 (25.9)
≥45	165 (17.0)	95 (15.9)	70 (18.7)
**Sex**			
Female	387 (39.8)	186 (31.2)	201 (53.6)
Male	585 (60.2)	411 (68.8)	174 (46.4)
**HIV status**			
Negative	774 (79.6)	434 (72.7)	340 (90.7)
Positive	198 (20.4)	163 (27.3)	35 (9.3)
**Education level**			
No/primary	806 (82.9)	500 (83.8)	306 (81.6)
Secondary/University	166 (17.1)	97 (16.2)	69 (18.4)
**Occupation**			
Unemployed	349 (35.9)	204 (34.2)	145 (38.7)
Employed	623 (64.1)	393 (65.8)	230 (61.3)
**Smoking status**			
No	831 (85.5)	489 (81.9)	342 (91.2)
Yes	141 (14.5)	108 (18.1)	33 (8.8)
**People in the household**			
≤3	731 (75.2)	442 (74.0)	289 (77.1)
>3	241 (24.8)	155 (26.0)	86 (22.9)
**Household income per month (US$)**			
≤100	763 (78.5)	473 (79.2)	290 (77.3)
>100	209 (21.5)	124 (20.8)	85 (22.7)
**Body weight at diagnosis, [in kg], (IQR)**	54 (48–61)	51 (46–57)	59 (53–67)
**BMI (kg/m^2^), median (IQR)**	20.0 (17.6–23.4)	18.3 (16.6–20.4)	23.9 (21.6–28.1)
**BMI categories (kg/m^2^)**			
Underweight <18.5	337 (34.7)	318 (53.3)	19 (5.1)
Normal, 18.5–24.9	454 (46.7)	256 (42.9)	198 (52.8)
Overweight 25.0–29.9	119 (12.2)	21 (3.5)	98 (26.1)
Obese ≥30	62 (6.4)	2 (0.3)	60 (16.0)
**Body fat (%)**	10.1 (7.7–14.7)	9.5 (6.8–13.7)	11.5 (8.5–17.0)
**MUAC (cm), median (IQR)**	24.3 (22.7–26.2)	23.3 (22.0–25.3)	25.3 (23.7–28.0)
**Waist hip ratio, median (IQR)**	0.89 (0.86–0.94)	0.89 (0.86–0.94)	0.89 (0.86–0.94)
**Occupational risk**^a^			
No	521 (54.2)	322 (54.2)	199 (54.1)
Yes	441 (45.8)	272 (45.8)	169 (45.9)
**Individual deworming (past 12 months)**			
Yes	797 (82.0)	484 (81.1)	313 (83.5)
No	175 (18.0)	113 (18.9)	62 (16.5)
**Hb level (g/dl), median (IQR)**	12 (10.4–13.3)	11.3 (9.9–12.7)	12.8 (11.5–14.1)

^a^ Occupational risk for acquiring schistosomiasis (working in rice fields, sand harvesting, washing cars, and fishing)

BMI, body mass index; HIV, human immunodefiency virus; Hb, hemoglobin level; IQR, inter-quartile range; MUAC, mid-upper arm circumference; US$, United States dollars (1 US$ = 2,190 Tanzanian Shillings in March 2016)

### Prevalence and risk factors for helminth infection

Among all participants, the prevalence of any helminth infection was 29.5% (95% CI: 26.7–32.6%). *S*. *stercoralis* (16.5%, 161) was the predominant helminth species, followed by hookworm (9.0%, 87), *S*. *mansoni* (5.7%, 55) and *S*. *haematobium* (2.0%, 19). Overall, TB patients were more frequently co-infected with any helminth species compared with controls (OR 1.34, 95% CI: 1.00–1.78, p = 0.048; Table 2). The prevalence of helminth infection was lower in HIV-positive (22.7%, 45) compared with HIV-negative study participants (31.3%, 242; S1 Table). Similarly, helminth infection was lower among TB patients co-infected with HIV (22.7%, 37) compared with HIV-negative TB patients (35.3%, 153; S2 Table). We found that most study participants had light-intensity helminth infection. For example, 96.4% (54) of study participants had light-intensity hookworm infection as determined by the Kato-Katz method (S3 Table). The prevalence and geographic distribution of species-specific helminth infections in the study area is shown in S1 Fig.

**Table 2:** Frequency distribution of helminth infections, stratified by TB patients and household contact controls.

Helminth infection	All	TB patients	Controls	Comparing TB patients and controls^a^	
	(n = 972)	(n = 597)	(n = 375)		
	n (%)	n (%)	n (%)	OR (95% CI)	p-value
**Any helminth**	287 (29.5)	190 (31.8)	97 (25.9)	1.34 (1.00–1.78)	0.048
**Helminth species**					
*Strongyloides stercoralis*	161 (16.6)	111 (18.6)	50 (13.3)	1.48 (1.03–2.13)	0.032
Hookworm	87 (9.0)	55 (9.2)	32 (8.5)	1.09 (0.69–1.72)	0.72
*Ascaris lumbricoides*	6 (0.6)	3 (0.5)	3 (0.8)	0.63 (0.13–3.12)	0.57
*Enterobius vermicularis*	5 (0.5)	1 (0.2)	4 (1.1)	NA	NA
*Trichuris trichiura*	9 (0.9)	6 (1.0)	3 (0.8)	1.25 (0.31–5.06)	0.75
*Hymenolepis diminuta*	2 (0.2)	1 (0.2)	1 (0.3)	NA	NA
*Schistosoma* spp.	70 (7.2)	49 (8.2)	21 (5.6)	1.51 (0.89–2.56)	0.13
*Schistosoma mansoni*	55 (5.7)	40 (6.7)	15 (4.0)	1.72 (0.94–3.17)	0.079
*Schistosoma haematobium*	19 (2.0)	11 (1.8)	8 (2.1)	0.86 (0.34–2.16)	0.75
**Helminth infection**					0.13
None	685 (70.5)	407 (68.2)	278 (74.1)	1	
Mono-infection	237 (24.4)	158 (26.5)	79 (21.1)	1.37 (1.00–1.86)	
Infection with ≥2 species	50 (5.1)	32 (5.3)	18 (4.8)	1.21 (0.67–2.21)	

^a^ Estimates from an unadjusted mixed-effect models with household as a random intercept

NA, not applicable; OR, odds ratio

Study participants with occupational risk for acquiring schistosomiasis, such as working in rice fields, sand harvesting, washing cars, and fishing had higher odds of being infected with any helminth species (aOR 1.42, 95% CI: 1.04–1.95, p = 0.029). HIV-positive patients were less likely to be infected with any helminth species (aOR 0.57, 95% CI: 0.37–0.87, p = 0.010; Table 3). Study participants who did not take anthelmintic treatment in the past 12 months did not have significant higher odds of being co-infected with any helminth species (aOR 1.35, 95% CI: 0.92–1.99, p = 0.12). There was no statistically significant interaction between the effects of HIV infection and deworming status on TB incidence (P-value from test for interaction: 0.5). When analyzing the risk factors for helminth infection separately for TB patients and household controls without TB, we found similar results (see S5 and S6 Tables).

**Table 3:** Risk factors for any helminth infection among TB patients and household controls without TB.

Characteristic	Helminth infection, n (%)		Unadjusted		Adjusted	
	Yes	No	OR (95% CI)	p-value	aOR (95% CI)	p-value
**Participant**				0.054		0.18
Controls	97 (33.8)	278 (40.6)	1.00		1.00	
TB patients	190 (66.2)	407 (59.4)	1.35 (1.00–1.82)		1.29 (0.88–1.87)	
**Age group (years)**				0.30		0.46
18–24	50 (17.4)	144 (21.0)	1.00		1.00	
25–34	115 (40.1)	232 (33.9)	1.46 (0.96–2.23)		1.38 (0.89–2.17)	
35–44	75 (26.1)	191 (27.9)	1.13 (0.72–1.78)		1.11 (0.68–1.82)	
≥45	47 (16.4)	118 (17.2)	1.16 (0.70–1.92)		1.18 (0.69–2.03)	
**Sex**				0.003		0.24
Female	93 (32.4)	294 (42.9)	1.00		1.00	
Male	194 (67.6)	391 (57.1)	1.60 (1.17–2.18)		1.23 (0.87–1.75)	
**HIV status**				0.022		0.010
Negative	242 (84.3)	532 (77.7)	1.00		1.00	
Positive	45 (15.7)	153 (22.3)	0.63 (0.43–0.94)		0.57 (0.37–0.87)	
**BMI category (kg/m^2^)**				0.077		0.47
BMI ≥18	175 (61.0)	460 (67.2)	1.00		1.00	
BMI <18	112 (39.0)	225 (32.8)	1.32 (0.97–1.79)		1.14 (0.79–1.64)	
**Education level**				0.28		0.50
No/primary	243 (84.7)	563 (82.2)	1.00		1.00	
Secondary/University	44 (15.3)	122 (17.8)	0.80 (0.53–1.20)		0.86 (0.55–1.34)	
**Employment status**				0.13		0.42
Unemployed	93 (32.4)	256 (37.4)	1.00		1.00	
Employed	194 (67.6)	429 (62.6)	1.28 (0.93–1.76)		1.16 (0.81–1.65)	
**Number of people in the household**				0.66		0.97
≤3	218 (76.0)	69 (24.0)	1.00		1.00	
>3	513 (74.9)	172 (25.1)	0.93 (0.65–1.32)		0.99 (0.69–1.42)	
**Household income per month (US$)**				0.47		0.75
≤100	229 (79.8)	534 (78.0)	1.00		1.00	
>100	58 (20.2)	151 (22.0)	0.87 (0.60–1.26)		0.94 (0.63–1.40)	
**Individual deworming (past 12 months)**				0.043		0.12
Yes	224 (78.0)	573 (83.6)	1.00		1.00	
No	63 (22.0)	112 (16.4)	1.48 (1.01–2.15)		1.35 (0.92–1.99)	
**Occupational risk**^a^				0.009		0.029
No	136 (47.7)	385 (56.9)	1.00		1.00	
Yes	149 (52.3)	292 (43.1)	1.50 (1.11–2.03)		1.42 (1.04–1.95)	

^a^ Occupational risk for acquiring schistosomiasis (working in rice fields, sand harvesting, washing cars, and fishing)

BMI, body mass index; HIV, human immunodeficieny virus; US$, United States dollars (1 US$ = 2,190 Tanzanian Shillings in March 2016)

Multilevel mixed-effects logistic regression model with household as a random intercept, adjusted for TB status, age-groups, sex, HIV status, BMI, education level, employment status, number of people living in the same household, individual deworming status, occupational risk, and income level.

Note: interaction between the effect of HIV and deworming status on the risk for any helminth infection: p = 0.50

### Helminth infection as a risk factor for TB

Multiple logistic regression models adjusted for patient characteristics and known risk factors for TB showed that any helminth infection was not statistically significantly associated with TB (aOR 1.26, 95% CI: 0.88–1.80, p = 0.22, Table 4 and S7 Table). However, when analyzing each helminth species separately, we found that *S*. *mansoni* infection was significantly associated with TB (aOR 2.15, 95% CI: 1.03–4.45, p = 0.040), but there was no significant association between TB and *S*. *stercoralis* or hookworm infection (S8 Table). Other co-factors that were significantly associated with TB included: male sex, HIV co-infection, smoking, living in a household with ≥3 people, and a low BMI (Table 4). The unadjusted and adjusted ORs for any helminth infection and *S*. *mansoni* are shown in S7 Table. Results were more pronounced when using a conditional logistic regression model (S9 Table).

**Table 4:** Associations of TB disease with helminth infection and other patient characteristics.

Characteristics			Any helminth infection (n = 972)		*S*. *mansoni* infection (n = 972)	
	TB patients	Controls	Adjusted		Adjusted	
	n (%)	n (%)	aOR (95% CI)	p-value	aOR (95% CI)	p-value
**Helminth infection**				0.22		0.040
No	407 (68.2)	278 (74.1)	1.00		1.00	
Yes	190 (31.8)	97 (25.9)	1.26 (0.88–1.80)		2.15 (1.03–4.45)	
**Age group (years)**				0.49		0.25
18–24	107 (17.9)	87 (23.2)	1.00		1.00	
25–34	226 (37.9)	121 (32.3)	1.22 (0.77–1.94)		1.24 (0.78–1.97)	
35–44	169 (28.3)	97 (25.9)	1.00 (0.60–1.67)		1.02 (0.61–1.7)	
≥45	95 (15.9)	70 (18.7)	0.85 (0.48–1.48)		0.88 (0.51–1.54)	
**Sex**				<0.001		<0.001
Female	186 (31.2)	201 (53.6)	1.00		1.00	
Male	411 (68.8)	174 (46.4)	3.12 (2.13–4.56)		3.16 (2.16–4.63)	
**HIV status**				<0.001		<0.001
Negative	434 (72.7)	340 (90.7)	1.00		1.00	
Positive	163 (27.3)	35 (9.3)	6.18 (3.83–9.95)		6.23 (3.86–10.05)	
**Education level**				0.55		0.57
No/primary	500 (83.8)	306 (81.6)	1.00		1.00	
Secondary/University	97 (16.2)	69 (18.4)	1.15 (0.73–1.80)		1.14 (0.72–1.79)	
**Employment status**				0.63		0.66
Unemployed	204 (34.2)	145 (38.7)	1.00		1.00	
Employed	393 (65.8)	230 (61.3)	0.91 (0.62–1.33)		0.92 (0.63–1.34)	
**Smoking status**				0.012		0.011
No	489 (81.9)	342 (91.2)	1.00		1.00	
Yes	108 (18.1)	33 (8.8)	1.92 (1.15–3.21)		1.95 (1.16–3.25)	
**Number of people in the household**				0.018		0.015
≤3 people	442 (74.0)	289 (77.1)	1.00		1.00	
>3 people	155 (26.0)	86 (22.9)	1.58 (1.08–2.30)		1.60 (1.09–2.34)	
**Household income per month (US$)**				0.85		0.95
≤100	473 (79.2)	290 (77.3)	1.00		1.00	
>100	124 (20.8)	85 (22.7)	1.04 (0.69–1.56)		1.01 (0.68–1.52)	
**BMI category (kg/m^2^)**				<0.001		<0.001
BMI ≥18	279 (46.7)	318 (53.3)	1.00		1.00	
BMI <18	356 (94.9)	19 (5.1)	23.20 (13.91–38.69)		23.52 (14.1–39.24)	
**Occupational risk**^a^				0.24		0.26
No	322 (54.2)	199 (54.1)	1.00		1.00	
Yes	272 (45.8)	169 (45.9)	0.82 (0.59–1.15)		0.83 (0.59–1.15)	
**Individual deworming (past 12 months)**				0.20		0.21
Yes	484 (81.1)	313 (83.5)	1.00		1.00	
No	113 (18.9)	62 (16.5)	0.75 (0.48–1.16)		0.76 (0.49–1.17)	

BMI, body mass index; CI, confidence interval; HIV, human immunodeficieny virus; OR, odds ratio; US$, United States dollars (1 US$ = 2,190 Tanzanian Shillings in March 2016)

^a^ Occupational risk for acquiring schistosomiasis (working in rice fields, sand harvesting, washing cars, and fishing)

Logistic regression model for TB disease status as the outcome. Model adjusted for any helminth infection/*S*. *mansoni*, age, sex, HIV status, BMI, education level, employment status, smoking status, number of people living in the same household, individual deworming status, helminth risk occupation and income level.

The full table with unadjusted and adjusted odds ratios is shown in the Supplementary Information (S7 Table).

### Effect of helminth infection on clinical presentation and disease severity in TB patients

TB patients co-infected with any helminth infection were more likely than helminth un-infected TB patients to present with hemoptysis (74 [38.9%] *vs*. 123 [30.2%]), had higher median hemoglobin levels (11.7 g/dl, IQR: 10.1–13.0 g/dl *vs*. 11.3 g/dl, IQR: 9.8–12.5 g/dl) and higher median eosinophil counts (0.2, IQR: 0.1–0.4 cells/μl *vs*. 0.1, IQR: 0.05–0.2 cells/μl; Table 5). TB patients co-infected with *S*. *mansoni* were more likely to have lower sputum bacterial load than helminth-uninfected TB patients (aOR 2.63; 95% CI: 1.38–5.26, p = 0.004). Furthermore, we found that TB patients co-infected with *S*. *mansoni* tended to have fewer lung cavities, although this association lacked statistical significance (aOR 0.41, 95% CI: 0.12–1.16, p = 0.088; Table 6). There were no statistically significant differences in radiological features between TB patients with and without any helminth infection as shown in S10 Table.

**Table 5:** Patient characteristics of TB patients infected and not infected with helminths at the time of TB diagnosis.

Characteristics	Total	TB and helminth	TB only	p-value
	(n = 597)	(n = 190)	(n = 407)	
**Age, median (IQR) (years)**	33 (26–40)	31 (26–39)	34 (27–40)	0.22
**Age groups (years)**				0.13
18–24	107 (17.9)	35 (18.4)	72 (17.7)	
25–34	226 (37.9)	81 (42.6)	145 (35.6)	
35–44	169 (28.3)	42 (22.1)	127 (31.2)	
≥45	95 (15.9)	32 (16.8)	63 (15.5)	
**Sex**				0.007
Female	186 (31.2)	45 (23.7)	141 (34.6)	
Male	411 (68.8)	145 (76.3)	266 (65.4)	
**HIV status**				0.003
Negative	434 (72.7)	153 (80.5)	281 (69.0)	
Positive	163 (27.3)	37 (19.5)	126 (31.0)	
**CD4^+^ count, cells/ml**^a^	202 (94–273)	185 (90–259)	203 (100–273)	0.74
**Education level**				0.49
No/primary	500 (83.8)	162 (85.3)	338 (83.0)	
Secondary/University	97 (16.2)	28 (14.7)	69 (17.0)	
**Occupation**				0.99
Unemployed	204 (34.2)	65 (34.2)	139 (34.2)	
Employed	393 (65.8)	125 (65.8)	268 (65.8)	
**Number of people in the household**				0.89
≤3 people	442 (74.0)	140 (73.7)	302 (74.2)	
> 3 people	155 (26.0)	50 (26.3)	105 (25.8)	
**Smoking status**				<0.004
No	489 (81.9)	143 (75.3)	346 (85.0)	
Yes	108 (18.1)	47 (24.7)	61 (15.0)	
**Household income per month (US$)**				0.45
≤100	473 (79.2)	154 (81.1)	319 (78.4)	
>100	124 (20.8)	36 (18.9)	88 (21.6)	
**Body weight (kg), median (IQR)**	51 (46–57)	50.9 (46–56)	51.7 (46–57.5)	0.40
**BMI (kg/m^2^), median(IQR)**	18.3 (16.6–20.4)	18.2 (16.5–20.2)	18.5 (16.6–20.4)	0.22
**BMI (kg/m^2^) groups, n (%)**				0.40^b^
Underweight <18.5	318 (53.3)	108 (56.8)	210 (51.6)	
Normal, 18.5–24.9	256 (42.9)	78 (41.1)	178 (43.7)	
Overweight 25.0–29.9	21 (3.5)	4 (2.1)	17 (4.2)	
Obese ≥30	2 (0.3)	0	2 (0.5)	
**Body fat (%)**	9.5 (6.8–13.7)	9.1 (6.0–12.7)	9.8 (7.4–14.0)	0.008
**MUAC (cm), median (IQR)**	23.3 (22.0–25.3)	23.7 (22.0–25.0)	23.3 (22.0–25.7)	0.99
**Waist hip ratio, median (IQR)**	0.89 (0.85–0.94)	0.89 (0.85–0.94)	0.89 (0.86–0.94)	0.75
**Occupational risk**				0.095
No	322 (54.2)	93 (49.2)	229 (56.5)	
Yes	272 (45.8)	96 (50.8)	176 (43.5)	
**Individual deworming (past 12 months)**				0.013
Yes	484 (81.1)	143 (75.3)	341 (83.8)	
No	113 (18.9)	47 (24.7)	66 (16.2)	
**Symptoms**^c^				
Cough	594 (99.5)	189 (99.5)	405 (99.5)	0.96
Fever	551 (92.3)	174 (91.6)	377 (92.6)	0.65
Weight loss	573 (96.0)	181 (95.3)	392 (96.3)	0.54
Night sweats	566 (94.8)	184 (96.8)	382 (93.9)	0.13
Hemoptysis	197 (33.0)	74 (38.9)	123 (30.2)	0.035
**TB score, median (IQR)**	5 (4–6)	5 (4–6)	5 (4–6)	0.22
TB score (0–5)	372 (62.3)	115 (60.5)	257 (63.1)	
TB score (6–12)	225 (37.7)	75 (39.5)	150 (36.9)	
**TB treatment categories**				0.40
Retreatment	14 (2.3)	3 (1.6)	11 (2.7)	
New patients	583 (97.7)	187 (98.4)	396 (97.3)	
**Blood parameters** ^c^				
Hemoglobin level	11.3 (9.9–12.7)	11.7 (10.1–13)	11.3 (9.8–12.5)	0.044
Eosinophil, cells per μl^d^	0.15 (0.06–0.32)	0.2 (0.1–0.4)	0.1 (0.05–0.2)	0.003

AFB, acid-fast bacilli; BMI, body mass index; HIV, human immunodefiency virus; IQR, interquartile range; MUAC, mid-upper arm circumference; US$, United States dollars (1 US$ = 2,190 Tanzanian Shillings in March 2016)

Helminth infection occupation risk (working in rice fields, sand harvesting, washing cars, and fishing)

^a^ TB patient co-infected with HIV and have CD4^+^ count values (n = 80)

^b^ Fisher’s exact test

^c^“Symptoms”, and “blood parameters”: categories not mutually exclusive

^d^ TB patients with an available full blood count result (n = 322)

**Table 6:** Effect of helminth infection on the clinical severity and clinical presentation in TB patients at the time of TB diagnosis.

Helminth infection	Severe TB score^a^		High sputum bacterial load^b^		Lung infiltration		Lung cavitation	
	aOR (95% CI)	p-value	aOR (95% CI)	p-value	aOR (95% CI)	p-value	aOR (95% CI)	p-value
**Any helminth infection**		0.55		0.12		0.42		0.82
No	1.00		1.00		1.00		1.00	
Yes	1.12 (0.78–1.61)		0.75 (0.51–1.08)		0.82 (0.50–1.33)		0.95 (0.60–1.50)	
***Strongyloides stercoralis***^c^		0.44		0.39		0.17		0.76
No	1.00		1.00		1.00		1.00	
Yes	1.19 (0.76–1.86)		0.82 (0.52–1.29)		1.56 (0.83–2.92)		1.09 (0.62–1.91)	
***Schistosoma mansoni*** ^d^		0.75		0.004		0.15		0.088
No	1.00		1.00		1.00		1.00	
Yes	0.89 (0.45–1.78)		0.37 (0.19–0.72)		0.51 (0.21–1.27)		0.41 (0.12–1.16)	
**Hookworm**^e^		0.55		0.40		0.086		0.54
No	1.00		1.00		1.00		1.00	
Yes	1.20 (0.67–2.15)		0.77 (0.42–1.42)		0.51 (0.23–1.10)		0.79 (0.37–1.69)	
**Multiple infections**		0.82		0.020		0.19		0.40
None	1.00		1.00		1.00		1.00	
Mono	1.13 (0.77–1.67)		0.88 (0.59–1.31)		0.85 (0.51–1.43)		1.06 (0.66–1.72)	
Double or more	1.04 (0.48–2.22)		0.34 (0.16–0.73)		0.67 (0.24–1.84)		0.50 (0.17–1.44)	

Logistic regression model adjusted for age, sex, HIV infection, and smoking status.

^a^ TB score (mild [score of 1–5] and severe [score of 6–12])

^b^ Sputum bacterial load (according to qualitative AFB smear microscopy grading): mild (scanty and 1+) and severe (≥+2)

^c^ 79 TB patients with any helminth infection other than *S*. *stercoralis* were excluded

^d^ 150 TB patients with helminth co-infection other than *S*. *mansoni* were excluded

^e^ 72 TB patients with helminth co-infection other than hookworm were excluded

### Effect of helminth infection on clinical outcomes in TB patients

Overall, 81.7% (273 TB patients) were cured at the end of TB treatment (at 6 months), 17.4% (58) completed treatment (AFB smear results not available at 6 months, but documented completion of treatment), and 0.9% (3) were treatment failures (positive AFB smear result at 6 months). We found no significant associations between helminth infection (at time of recruitment) and poor gain in absolute weight (aOR 0.89, 95% CI: 0.55–1.45, p = 0.63), BMI (aOR 0.74, 95% CI: 0.46–1.21, p = 0.23), and body fat percentage (aOR 0.92, 95% CI: 0.55–1.56, p = 0.78) after 6 months on TB treatment, as shown in S11 Table.

## Discussion

We present findings on the prevalence and association of TB and helminth co-infection among adult TB patients and household contact controls in a highly-urbanized setting of Dar es Salaam, Tanzania. We found that *S*. *mansoni* infection was a risk factor for TB disease. This association remained significant after adjustment for other known risk factors for TB, such as HIV infection, smoking, and underweight [35]. None of the other investigated helminth species or the surrogate measure of “any helminth infection” were significantly associated with TB. Importantly, associations between any helminth co-infection and TB were reported in previous epidemiologic studies [21,36,37], as well as in experimental work using animal or macrophage infection models [9,13,15]. In line with our findings, a recent study with human peripheral mononuclear cells exposed to *M*. *tuberculosis* and *S*. *mansoni* antigens showed that *S*. *mansoni*-induced CD4^+^ T cells disrupt the control of *M*. *tuberculosis* in infected macrophages [9].

Several studies in humans suggested that helminth infections may increase the risk for progression of latent *M*. *tuberculosis* infection to active TB [15,21,37] as well as for exacerbating the disease [15]. However, the results of these studies are conflicting, and no differentiation at the helminth species level was made in these analyses. Indeed, the hypothesis of a helminth species-specific impact on the host response is supported by a recent systematic review, which revealed a trend toward an association between a decrease in HIV viral loads and treatment for *S*. *mansoni*, but not for other helminth species [38]. A case-control study from Ethiopia also found an association between TB and helminth infections, and the association was stronger in patients that were infected with multiple helminth species [21]. The small number of study participants with *S*. *mansoni* infection (31 among TB cases, nine among controls) may have masked an association between TB and schistosomiasis in that study [21]. In contrast, a cohort study from India showed no difference in TB incidence rates in helminth-infected and helminth-free individuals after 2.5 years of follow-up [39].

We also found that *S*. *mansoni*, but not other helminth species, was associated with the clinical presentation among TB patients. Patients co-infected with *S*. *mansoni* had lower sputum bacterial loads at the time of TB diagnosis than *S*. *mansoni*-negative TB patients. Similarly, a study in Ethiopia observed lower sputum bacterial loads at TB diagnosis in TB patients co-infected with any helminth species [40]. Interestingly, our observation in TB patients co-infected with *S*. *mansoni* resembles the paucibacillary disease in HIV-positive individuals with severe immunosuppression, who frequently have negative or low bacterial *M*. *tuberculosis* loads in the sputum compared with HIV-negative patients [40,41]. Hence, the helminth-induced Th1 immunological impairment might have an effect on the sputum bacterial load. Moreover, TB patients with an impaired host immune system rarely present with lung cavitation resulting in fewer *M*. *tuberculosis* bacilli being expectorated in the sputum [40,41]. This is in line with our findings that TB patients co-infected with *S*. *mansoni* tended to present less frequently with lung cavitations compared with *S*. *mansoni*-negative TB patients. Any helminth co-infection did not appear to have an effect on clinical outcomes during follow-up. We found no evidence for an effect of helminth co-infection on the gain in the percentage of body fat and BMI after 6 months (e.g., at the time of completed TB treatment). This might be explained by the fact that the administration of anthelmintic treatment offered to the study participants after diagnosis might have reversed the Th1 immune response [15], and thus attenuated the effect of helminth infections on clinical outcomes. However, the effect of a reversal of the Th1 immune response could be minimal as the anthelmintic drugs target the worms [42], which are less immunogenic compared with deposited *S*. *mansoni* eggs [9].

We found that TB patients had a higher crude prevalence of helminth infections, as compared with household contact controls. The higher prevalence of helminth infections among TB patients could be the result of the pathogenic role of helminth infection in the progression from *M*. *tuberculosis* infection to active TB. The higher prevalence of helminth co-infection in TB patients has also been noted in other studies from different settings [9,43]. For example, a study conducted in Ethiopia reported a higher prevalence of helminth infection among TB patients as compared with household contact controls [21]. Overall, the prevalence of helminth infection in our study was 32% and lower compared with the 71% observed in the latter study [21]. It is conceivable that the high proportion of self-reported previous use of anthelmintic drugs in our study (approximately 80%) could have reduced the overall prevalence of helminth infection. Hence, we may have underestimated the effects of helminth infection seen in our study.

We also found that occupation exposing people to regular water contacts (for instance rice field workers, sand harvesters, car washers, and fishermen) were associated with helminth infections. Being exposed to freshwater bodies and being involved in water-related activities have previously been reported to increase the risk of helminth infections [44]. In the current study, HIV-positive individuals were less likely to be co-infected with helminths. A lower prevalence of helminth infections in HIV-positive patients has also been reported in a study conducted in Mwanza in northern Tanzania, which is a highly endemic area for helminthiases [45]. Of note, current clinical practice in Tanzania is to treat any helminth infection in HIV-positive patients at enrolment into HIV care and in case of clinical suspicion of helminth infection during follow-up, as specified in the HIV/AIDS management guideline [17]. The use of anthelmintic drugs is safe and might be beneficial in HIV-positive patients by possibly reducing the HIV-RNA viral load and subsequently improving clinical outcomes [46]. Furthermore, cotrimoxazole preventive therapy (CPT), which is recommended for HIV-positive patients, has also been reported to have limited anthelmintic properties [43,47]. This might explain the lower prevalence of helminth infection among HIV-positive individuals in our study [17].

Our research has several strengths and limitations that warrant consideration. An important strength of our study is the large sample size and the recruitment of both TB patients and household contact controls with similar socioeconomic profiles and exposure patterns to both TB and helminth infection. Our findings may well apply to other settings with a similar prevalence of TB, HIV, and helminth infections in sub-Saharan Africa. Furthermore, we used recommended TB diagnostics and a suite of standardized, quality-controlled helminth diagnostics, which have comparable diagnostic performance to resource-intensive molecular test assays [25].

Study limitations include the following. First, this is an observational study which cannot establish a causal relationship between helminth infections and TB disease. Second, we could not fully verify whether or not the household contact controls were latently infected with *M*. *tuberculosis*, which is a prerequisite to develop TB. However, because Dar es Salaam is a high-burden setting for TB with considerable risk of transmission, and because living with a TB patient is a strong risk factor for TB [35], it is reasonable to assume that the controls have previously been exposed and infected with *M*. *tuberculosis*. Third, we did not check the helminth infection status for TB patients during and after completion of TB treatment, which could influence the clinical outcomes. However, we do not expect a high helminth re-infection rate after 6 months in our study area [48]. Fourth, we did not use molecular diagnostics such as polymerase chain reaction (PCR) which might have identified some more cases, but one of our previous studies revealed that also PCR approaches miss in particular very light intensity infections. Moreover, its performance and sensitivity vary with the helminth species under examination [25]. Hence, also a PCR cannot be considered as the diagnostic gold-standard.

In conclusion, co-infection with *S*. *mansoni*, but not other helminth species, was found to be an independent risk factor for active TB in our study and was associated with the clinical presentation in TB patients. These findings suggest a role for *S*. *mansoni*, or helminth infection in general, in immunomodulation of human TB. Treatment of helminth infections should be considered in the clinical management of TB patients, and helminthiasis control/elimination through preventive chemotherapy might prove to be useful as an additional component of TB control programs. Further research is needed to establish the underlying mechanisms, and compare helminth-induced immune regulation by different helminth species. Prospective cohort studies that evaluate the effect of preventive anthelmintic chemotherapy on the incidence of *M*. *tuberculosis* infection and active TB could further help to understand the interaction between these diseases at the population level. Helminthiasis control measures, in combination with traditional TB control strategies, could potentially contribute to the global efforts to reduce TB incidence by 80% until 2030, as stipulated in WHO’s ambitious End TB Strategy [5].

## Supporting information

S1 Table Socio-demographic and clinical characteristics of TB patients and household contact controls, stratified by HIV infection status. (DOCX)

S2 Table Frequency distribution of helminth infections among TB patients and household controls without TB, stratified by HIV status. (DOCX)

S3 Table Frequency distribution and intensity of helminth infection in TB patients and household contact controls, as determined by the Kato-Katz method (triplicate slides). (DOCX)

S4 Table Full blood count and hematological parameters in TB patients, stratified by helminth infection status. (DOCX)

S5 Table Additional analysis: Risk factors for any helminth infection among TB patients only. (DOCX)

S6 Table Additional analysis: Risk factors for any helminth infection among household controls without TB only. (DOCX)

S7 Table Full table with unadjusted and adjusted odds ratios. Associations of TB disease with helminth infection and other patient characteristics comparing TB patients and household contact controls without TB.
(DOCX)

S8 Table Associations of TB disease with *Strongyloides stercoralis* and hookworm infection(unadjusted and adjusted odds ratios). (DOCX)

S9 Table Additional analysis: Helminth infection and patient characteristics associated with TB among TB patients and household contact controls, using conditional logistic regression. (DOCX)

S10 Table Radiological findings of chest X-rays in TB patients at the time of TB diagnosis, stratified by helminth infection status. (DOCX)

S11 Table Association of helminth infection with poor recovery of BMI, poor gain of absolute weight, and percentage body fat in TB patients, between recruitment and after 6 months of completed TB treatment. (DOCX)

S1 Fig Geographic distribution of helminth infection in the study area. (A) The prevalence of helminth infection summarized at the ward level. (B) The helminth species distribution at the study area. Other helminth infections include: *Ascaris lumbricoides*, *Enterobius vermicularis*, *Trichuris trichiura*, and *Hymenolepis diminuta*.
(DOCX)

S1 Checklist STROBE checklist completed for this manuscript. (DOCX)
