# Supplementary material for: Prevalence and clinical relevance of helminth co-infections among tuberculosis patients in urban Tanzania
Source: PLoS Negl Trop Dis. 2017 Feb 8;11(2):e0005342. doi: 10.1371/journal.pntd.0005342 (PMC5319816; doi:10.1371/journal.pntd.0005342)
Supplement: S1 Table — (DOCX) [file pntd.0005342.s001.docx]

**Title: Prevalence and Clinical Relevance of Helminth Co-infections among Tuberculosis Patients in Urban Tanzania**

**S1 Table. Socio-demographic and clinical characteristics of TB patients and household contact controls without TB, stratified by HIV infections status.**

| Characteristics | Total |  | HIV status | |
| --- | --- | --- | --- | --- |
|  | (n=972) |  | **Positive** (n=198) | **Negative** (n=774) |
| Type of study participant |  |  |  |  |
| Control | 375 (38.6) |  | 35 (17.7) | 340 (43.9) |
| Case | 597 (61.4) |  | 163 (82.3) | 434 (56.1) |
| Helminth status |  |  |  |  |
| Negative | 685 (70.5) |  | 153 (77.3) | 532 (68.7) |
| Positive | 287 (29.5) |  | 45 (22.7) | 242 (31.3) |
| Age, median (IQR), years | 33 (26-41) |  | 38 (33-44) | 38 (25-39) |
| Age groups (years) |  |  |  |  |
| 18-24 | 194 (20.0) |  | 6 (3.0) | 188 (24.3) |
| 25-34 | 347 (35.7) |  | 64 (32.3) | 283 (36.6) |
| 35-44 | 266 (27.4) |  | 82 (41.4) | 184 (23.8) |
| ≥45 | 165 (17.0) |  | 46 (23.2) | 119 (15.4) |
| Sex |  |  |  |  |
| Female | 387 (39.8) |  | 105 (53.0) | 282 (36.4) |
| Male | 585 (60.2) |  | 93 (47.0) | 492 (63.6) |
| Education level |  |  |  |  |
| No formal education & Primary | 806 (82.9) |  | 185 (93.4) | 621 (80.2) |
| Secondary/University | 166 (17.1) |  | 13 (6.6) | 153 (19.8) |
| Occupation |  |  |  |  |
| Unemployed | 349 (35.9) |  | 62 (31.3) | 287 (37.1) |
| Employed | 623 (64.1) |  | 136 (68.7) | 487 (62.9) |
| Household income (USD) |  |  |  |  |
| ≤100 | 763 (78.5) |  | 154 (77.8) | 609 (78.7) |
| >100 | 209 (21.5) |  | 44 (22.2) | 165 (21.3) |
| Weight (kg), median(IQR) | 54 (48-61) |  | 53 (47.0-59.7) | 54.4 (49.0-61.0) |
| BMI (kg/m^2^), median(IQR) | 20.0 (17.6-23.4) |  | 19.3 (17.2-22.1) | 20.3 (17.8-23.9) |
| BMI categories (kg/m^2^) |  |  |  |  |
| Underweight, <18.5 | 337 (34.7) |  | 81 (40.9) | 256 (33.1) |
| Normal, 18.5-24.9 | 454 (46.7) |  | 96 (48.5) | 358 (46.3) |
| Overweight, 25.0-29.9 | 119 (12.2) |  | 14 (7.1) | 105 (13.6) |
| Obese, ≥30 | 62 (6.4) |  | 7 (3.5) | 55 (7.1) |
| Body fat percentage (%), median (IQR) | 10.1 (7.7-14.7) |  | 12.2 (8.7-15.8) | 9.7 (7.2-14.4) |
| MUAC (cm), median (IQR) | 24.3 (22.6-26.1) |  | 23.6 (22.0-25.7) | 24.3 (22.7-26.3) |
| Waist hip ratio, median (IQR) | 0.89 (0.86-0.94) |  | 0.89 (0.86-0.93) | 0.89 (0.86-0.94) |
| Risk occupation |  |  |  |  |
| No | 521 (54.2) |  | 102 (51.5) | 429 (55.4) |
| Yes | 441 (45.8) |  | 96 (48.5) | 345 (44.6) |
| Individual deworming |  |  |  |  |
| Dewormed within 12months | 797 (82.0) |  | 162 (81.8) | 635 (82.0) |
| Never within 12 months | 175 (18.0) |  | 36 (18.2) | 139 (18.0) |
| Hemoglobin level (g/dL), median (IQR) | 12.0 (10.4-13.3) |  | 10.4 (9.0-11.9) | 12.3 (10.9-13.6) |
| Anemia status |  |  |  |  |
| Normal (Hb ≥11 g/dL) | 657 (67.6) |  | 82 (41.4) | 575 (74.3) |
| Anaemia (Hb <11 g/dL) | 315 (32.4) |  | 116 (58.6) | 199 (25.7) |

IQR, inter-quartile range; BMI, body mass index; HIV, human immunodefiency Virus; MUAC, Mid-upper arm circumference; Tshs, Tanzanian Shillings; risk occupation for helminth infection (rice fields, car washing, rice harvesting and fishing)
