# Supplementary material for: Prevalence and clinical relevance of helminth co-infections among tuberculosis patients in urban Tanzania
Source: PLoS Negl Trop Dis. 2017 Feb 8;11(2):e0005342. doi: 10.1371/journal.pntd.0005342 (PMC5319816; doi:10.1371/journal.pntd.0005342)
Supplement: S2 Table — (DOCX) [file pntd.0005342.s002.docx]

**Title: Prevalence and Clinical Relevance of Helminth Co-infections among Tuberculosis Patients in Urban Tanzania**

**S2 Table. Frequency distribution of helminth infections among TB patients and household controls without TB, stratified by HIV status.**

| Helminth Infection | All (n=972) | TB patients ((n=597) | | |  | Controls (n=375) | | |
| --- | --- | --- | --- | --- | --- | --- | --- | --- |
|  |  | HIV-positive | HIV-negative | p-value |  | HIV-positive | HIV-negative | p-value^a^ |
|  |  | (n=163) | (n=434) |  |  | (n=35) | (n=340) |  |
| Any helminth | 287 (29.5) | 37 (22.7) | 153 (35.3) | 0.003 |  | 8 (22.9) | 89 (26.2) | 0.67 |
|  |  |  |  |  |  |  |  |  |
| Helminth species |  |  |  |  |  |  |  |  |
| *Strongyloides stercolaris* | 161 (16.6) | 19 (11.7) | 92 (21.2) | 0.008 |  | 7 (20.0) | 43 (12.6) | 0.22 |
| Hookworm | 87 (9.0) | 7 (4.3) | 48 (11.1) | 0.011 |  | 1 (2.9) | 31 (9.1) | 0.21 |
| *Ascaris lumbricoides* | 6 (0.6) | 1 (0.6) | 2 (0.5) | 0.62^a^ |  | 0 (0) | 3 (0.9) | 0.75 |
| *Enterobius vermicularis* | 5 (0.5) | 0 (0) | 1 (0.2) | 0.73 ^a^ |  | 1 (2.9) | 3 (0.9) | 0.33 |
| *Trichuris trichiura* | 9 (0.9) | 4 (2.5) | 2 (0.5) | 0.050 ^a^ |  | 0 (0) | 3 (0.9) | 0.75 |
| *Hymenolepis dimunita* | 2 (0.2) | 0 (0) | 1 (0.2) | 0.73^a^ |  | 0 (0) | 1 (0.3) | 0.91 |
| Schistosoma spp.^b^ | 70 (7.2) | 10 (6.1) | 39 (9.0) | 0.26 |  | 0 (0) | 15 (4.4) | 0.13 |
| *Schistosoma mansoni* | 55 (5.7) | 10 (6.1) | 30 (6.9) | 0.74 |  | 0 (0) | 15 (4.4) | 0.22 |
| *Schistosoma hematobium* | 19 (2.0) | 1 (0.6) | 10 (2.3) | 0.15^a^ |  | 0 (0) | 8 (2.4) | 0.36 |
|  |  |  |  |  |  |  |  |  |
| Multiple helminth infection |  |  |  | 0.016 |  |  |  | 1.000 |
| Mono-infection | 237 (24.4) | 33 (20.2) | 125 (28.8) |  |  | 7 (20) | 20 (79) |  |
| Double infection | 44 (4.5) | 3 (1.8) | 24 (5.5) |  |  | 1 (2.9) | 2.9 (17) |  |
| Triple infection | 6 (0.6) | 1 (0.6) | 4 (0.9) |  |  | 0 (0) | 0 (1) |  |

^a^Fisher’s exact; ^b^ Four patients were co-infected with both *Schistosoma mansoni* and *Schistosoma hematobium*
