# Supplementary material for: Prevalence and clinical relevance of helminth co-infections among tuberculosis patients in urban Tanzania
Source: PLoS Negl Trop Dis. 2017 Feb 8;11(2):e0005342. doi: 10.1371/journal.pntd.0005342 (PMC5319816; doi:10.1371/journal.pntd.0005342)
Supplement: S3 Table — (DOCX) [file pntd.0005342.s003.docx]

**Title: Prevalence and Clinical Relevance of Helminth Co-infections among Tuberculosis Patients in Urban Tanzania**

**S3 Table. Frequency distribution and intensity of helminth infection in TB patients and household contact controls, as determined by the Kato-Katz method (triplicate slides).**

| Helminth infection ^a^ | All | TB patients | Controls | p-value |
| --- | --- | --- | --- | --- |
| n (%) | (n=89) | (n=54) | (n=35) |  |
|  |  |  |  |  |
| Hookworm | 56 (5.8) | 35 (5.9) | 21 (5.6) | NA |
| EPG, median (IQR) | 120 (40-480) | 104 (32-336) | 249 (64-560) | 0.16^b^ |
| Infection intensity (epg) |  |  |  | NA |
| Light (1-1,999) | 54 (96.4) | 35 (100) | 19 (90.5) |  |
| Moderate (2,000-3,999) | 1 (1.8) | 0 | 1 (4.8) |  |
| Severe (≥4,000) | 1 (1.8) | 0 | 1 (4.8) |  |
|  |  |  |  |  |
| *Schistosoma mansoni* | 25 (2.6) | 14 (2.3) | 11 (2.9) | NA |
| EPG, median (IQR) | 56 (16-88) | 52 (16-88) | 56 (16-88) | 0.83^b^ |
| Infection intensity (epg) |  |  |  |  |
| Light (1-99) | 20 (80.0) | 11 (78.6) | 9 (81.8) | NA |
| Moderate (100-399) | 4 (16.0) | 3 (21.4) | 1 (9.1) |  |
| Severe (≥400) | 1 (4.0) | 0 | 1 (9.1) |  |
|  |  |  |  |  |
| *Trichuris trichiura* | 8 (0.8) | 6 (1.0) | 2 (0.5) | NA |
| EPG, median (IQR) | 24 (12-80) | 24 (16-120) | 24 (8-40) | 0.61^b^ |
| Infection intensity (epg) |  |  |  |  |
| Light (1-999) | 8 (100) | 6 (100) | 2 (100) | NA |
|  |  |  |  |  |
| *Ascaris lumbricoides* | 3 (0.3) | 1 (0.2) | 2 (0.5) | NA |
| EPG, median (IQR) | 16 (16-80) | 80 (80-80) | 16 (16-16) | 0.16^b^ |
| Infection intensity (epg) |  |  |  |  |
| Light (1-5,000) | 3 (100) | 1 (100) | 2 /100) |  |

^a^ Four patients were co-infected by *Schistosoma mansoni* and *Schistosoma haematobium*;

^b^ Wilcoxon signed rank test; Helminth infection risk occupation (rice fields, car wash, rice harvest and fishing); IQR, inter-quartile range; EPG, Eggs per gram; NA, Not Applicable

**^x^ Helminth intensity infection: Light**: *A. lumbricoides*, 1-4,999 EPG; *T. trichiura*, 1-999 EPG; hookworms, 1-1,999 EPG; and *S. mansoni*, 1-99 EPG. **Moderate**: *A. lumbricoides*, 5,000-49,999 EPG; *T. trichiura*, 1,000-9,999 EPG; hookworms, 2,000-3,999 EPG; and *S. mansoni*, 100-399 EPG. **Severe:** *A. lumbricoides*, ≥50,000 EPG; *T. trichiura* ≥10,000 EPG; hookworms, ≥4,000 EPG and *S. mansoni*, ≥400 EPG
