# Supplementary material for: Prevalence and clinical relevance of helminth co-infections among tuberculosis patients in urban Tanzania
Source: PLoS Negl Trop Dis. 2017 Feb 8;11(2):e0005342. doi: 10.1371/journal.pntd.0005342 (PMC5319816; doi:10.1371/journal.pntd.0005342)
Supplement: S4 Table — (DOCX) [file pntd.0005342.s004.docx]

**Title: Prevalence and Clinical Relevance of Helminth Co-infections among Tuberculosis Patients in Urban Tanzania**

**S4 Table. Full blood count and hematological parameters in TB patients, stratified by helminth infection status.**

| Blood parameters | Total | Helminth status | | p-value^b^ |
| --- | --- | --- | --- | --- |
|  | (n=566) ^a^ | **Positive** (n=179) | **Negative** (n=387) |  |
|  |  |  |  |  |
| White blood cells (×10^9^/L) | 7.4 (5.7-9.6) | 7.3 (5.6-9.3) | 7.5 (5.7-9.8) | 0.48 |
| Red blood cells (×10^9^/L) | 4.7 (4.1-5.29 | 4.7 (4.3-5.4) | 4.6 (4.0-5.2) | 0.11 |
| Haemoglobin level (g/dL) | 11.2 (9.7-12-6) | 11.5 (10.2-13) | 11.1 (9.5-12.5) | 0.014 |
| Haematocrit (%) | 34.8 (30.5-38.9) | 35.9 (31.6-39.6) | 34.6 (30.0-38.3) | 0.021 |
| MCV (fL) | 75.9 (68.9-82.4) | 76.0 (70.3-81.6) | 75.6 (68.3-83) | 0.97 |
| MCH (pg) | 24.3 (21.6-26.7) | 24.3 (22-27) | 24.2 (21.5-26.7) | 0.47 |
| MCHC (%) | 32.4 (30.6-33-6) | 32.5 (30.9-33.7) | 32.3 (30.4-33.5) | 0.20 |
| Platelets (×10^9^/L) | 350 (263-460) | 366 (247-430) | 350 (263-460) | 0.29 |
|  |  |  |  |  |
| Differential blood counts (×10^9^/L) | (n=332) | (n=112) | (n=220) |  |
| Neuotrophil | 4.7 (3.3-6.3) | 4.6 (3.1-6.1) | 4.8 (3.4-6.4) | 0.39 |
| Lymphocyte | 1.5 (1.2-2.0) | 1.6 (1.2-2.0) | 1.5 (1.2-2.1) | 0.46 |
| Monoctye | 0.77 (0.55-1.0) | 0.8 (0.5-1.1) | 0.8 /0.6-1.0) | 0.86 |
| Eosinophil | 0.15 (0.06-0.32) | 0.2 (0.1-0.4) | 0.1 (0.05-0.2) | 0.003 |
| Basophil | 0.03 (0.02-0.05) | 0.03 (0.02-0.07) | 0.03 (0.02-0.05) | 0.30 |

MCH, mean corpuscular hemoglobin; MCHC, mean corpuscular hemoglobin concentration; MCV, mean corpuscular volume

^a^ 31 TB patients were excluded from the analysis because of the missing full blood count results;

^b^Wilcoxon rank-sum test; all measurements are in median (Interquartile Range, [IQR])

^c^ 244 TB patients had missing differential count of white blood counts
