# Supplementary material for: Prevalence and clinical relevance of helminth co-infections among tuberculosis patients in urban Tanzania
Source: PLoS Negl Trop Dis. 2017 Feb 8;11(2):e0005342. doi: 10.1371/journal.pntd.0005342 (PMC5319816; doi:10.1371/journal.pntd.0005342)
Supplement: S5 Table — (DOCX) [file pntd.0005342.s005.docx]

**Title: Prevalence and Clinical Relevance of Helminth Co-infections among Tuberculosis Patients in Urban Tanzania**

**S5 Table. Additional analysis: risk factors for any helminth infection among TB patients only.**

| Characteristics | Helminth Infection | | Crude |  |  | Adjusted |  |
| --- | --- | --- | --- | --- | --- | --- | --- |
| n (%) | Yes | No |  |  |  |  |  |
|  | n (%) | (n (%) | OR (95% CI) | p-value |  | aOR (95% CI) | p-value |
| Age group (years) |  |  |  | 0.13 |  |  | 0.095 |
| 18-24 | 35 (18.4) | 72 (17.7) | 1.00 |  |  | 1.00 |  |
| 25-34 | 81 (42.6) | 145 (35.6) | 1.15 (0.71-1.87) |  |  | 1.25 (0.74-2.11) |  |
| 35-44 | 42 (22.1) | 127 (31.2) | 0.68 (0.40-1.16) |  |  | 0.70 (0.38-1.26) |  |
| ≥45 | 32 (16.8) | 63 (15.5) | 1.04 (0.58-1.88) |  |  | 1.11 (0.58-2.11) |  |
| Sex |  |  |  | 0.006 |  |  | 0.058 |
| Female | 45 (23.7) | 141 (34.6) | 1.00 |  |  | 1.00 |  |
| Male | 145 (76.3) | 266 (65.4) | 1.71 (1.15-2.53) |  |  | 1.53 (0.99-2.37) |  |
| HIV status |  |  |  | 0.003 |  |  | 0.038 |
| Negative | 153 (80.5) | 281 (69.0) | 1.00 |  |  | 1.00 |  |
| Positive | 37 (19.5) | 126 (31.0) | 0.534 (0.36-0.82) |  |  | 0.62 (0.39-0.97) |  |
| Education level |  |  |  | 0.49 |  |  | 0.45 |
| No/primary | 162 (85.3) | 338 (83.0) | 1.00 |  |  | 1.00 |  |
| Secondary/University | 28 (14.7) | 69 (17.0) | 0.85 (0.53-1.37) |  |  | 0.81 (0.48-1.38) |  |
| Employment status |  |  |  | 0.99 |  |  | 0.56 |
| Unemployed | 65 (34.2) | 139 (34.2) | 1.00 |  |  | 1.00 |  |
| Employed | 125 (65.8) | 268 (65.8) | 1.00 (0.69-1.43) |  |  | 0.89 (0.59-1.33) |  |
| People in the household |  |  |  | 0.89 |  |  | 0.49 |
| ≤3 people | 140 (73.7) | 302 (74.2) | 1.00 |  |  | 1.00 |  |
| > 3 people | 50 (26.3) | 105 (25.8) | 1.03 (0.69-1.52) |  |  | 1.16 (0.77-1.74) |  |
| Household income (USD) |  |  |  | 0.45 |  |  | 0.96 |
| ≤100 | 154 (81.1) | 319 (78.4) | 1.00 |  |  | 1.00 |  |
| >100 | 36 (18.9) | 88 (21.6) | 0.85 (0.55-1.31) |  |  | 1.01 (0.63-1.62) |  |
| BMI category (kg/m^2^) |  |  |  | 0.23 |  |  | 0.38 |
| BMI ≥18 | 82 (43.2) | 197 (48.4) | 1.00 |  |  | 1.00 |  |
| BMI < 18 | 108 (56.8) | 210 (51.6) | 1.24 (0.87-1.75) |  |  | 1.17 (0.82-1.69) |  |
| Occupational risk |  |  |  | 0.096 |  |  | 0.14 |
| No | 93 (49.2) | 229 (56.5) | 1.00 |  |  | 1.00 |  |
| Yes | 96 (50.8) | 176 (43.5) | 1.34 (0.95-1.89) |  |  | 1.32 (0.91-1.90) |  |
| Individual deworming (in 12 months) |  |  |  | 0.015 |  |  | 0.022 |
| Yes | 143 (75.3) | 341 (83.8) | 1.00 |  |  | 1.00 |  |
| No | 47 (24.7) | 66 (16.2) | 1.70 (1.11-2.59) |  |  | 1.67 (1.08-2.60) |  |

BMI, body mass index; HIV, human immunodeficieny virus; Helminth infection risk occupation (working in the rice fields, car wash, rice harvest and fishing)

Logistic regression model was used. including the independent variables TB status, age-group, sex, HIV status, BMI, education level, employment status, number of people living in the same household, individual deworming status, occupational risk and income level in quartiles.  
