# Supplementary material for: Prevalence and clinical relevance of helminth co-infections among tuberculosis patients in urban Tanzania
Source: PLoS Negl Trop Dis. 2017 Feb 8;11(2):e0005342. doi: 10.1371/journal.pntd.0005342 (PMC5319816; doi:10.1371/journal.pntd.0005342)
Supplement: S6 Table — (DOCX) [file pntd.0005342.s006.docx]

**SUPPORTING INFORMATION**

**Title: Prevalence and Clinical Relevance of Helminth Co-infections among Tuberculosis Patients in Urban Tanzania**

**S6 Table. Additional analysis: risk factors for any helminth infection among household controls without TB only.**

| Characteristic | Helminth status | |  | Crude |  |  | Adjusted |  |
| --- | --- | --- | --- | --- | --- | --- | --- | --- |
|  | Yes | No |  |  |  |  |  |  |
|  | n (%) | (n (%) |  | OR (95% CI) | p-value |  | aOR (95% CI) | p-value |
| Age group (years) |  |  |  |  | 0.055 |  |  | 0.091 |
| 18-24 | 15 (15.5) | 72 (25.9) |  | 1.00 |  |  | 1.00 |  |
| 25-34 | 34 (35.1) | 87 (31.3) |  | 1.88 (0.95-3.71) |  |  | 1.70 (0.82-3.50) |  |
| 35-44 | 33 (34.0) | 64 (23.0) |  | 2.47 (1.23-4.97) |  |  | 2.52 (1.17-5.47) |  |
| ≥45 | 15 (15.5) | 55 (19.8) |  | 1.31 (0.59-2.91) |  |  | 1.29 (0.55-3.02) |  |
| Sex |  |  |  |  | 0.35 |  |  | 0.80 |
| Female | 48 (49.5) | 153 (55.0) |  | 1.00 |  |  | 1.00 |  |
| Male | 49 (50.5) | 125 (45.0) |  | 1.25 (0.79-1.99) |  |  | 0.93 (0.55-1.59) |  |
| HIV status |  |  |  |  | 0.67 |  |  | 0.35 |
| Negative | 89 (91.8) | 251 (90.3) |  | 1.00 |  |  | 1.00 |  |
| Positive | 8 (8.2) | 27 (9.7) |  | 0.84 (0.37-1.91) |  |  | 0.66 (0.28-1.57) |  |
| Education level |  |  |  |  | 0.57 |  |  | 0.82 |
| No/primary | 81 (83.5) | 225 (80.9) |  | 1.00 |  |  | 1.00 |  |
| Secondary/University | 16 (16.5) | 53 (19.1) |  | 0.84 (0.45-1.55) |  |  | 1.08 (0.54-2.18) |  |
| Employment status |  |  |  |  | 0.020 |  |  | 0.099 |
| Unemployed | 28 (28.9) | 117 (42.1) |  | 1.00 |  |  | 1.00 |  |
| Employed | 69 (71.1) | 161 (57.9) |  | 1.79 (1.09-2.95) |  |  | 1.63 (0.91-2.92) |  |
| People in the household |  |  |  |  | 0.36 |  |  | 0.63 |
| ≤3 people | 78 (80.4) | 211 (75.9) |  | 1.00 |  |  | 1.00 |  |
| > 3 people | 19 (19.6) | 67 (24.1) |  | 0.77 (0.43-1.36) |  |  | 0.86 (0.47-1.58) |  |
| Household income (USD) |  |  |  |  | 0.99 |  |  | 0.37 |
| ≤100 | 75 (77.3) | 215 (77.3) |  | 1.00 |  |  | 1.00 |  |
| >100 | 22 (22.7) | 63 (22.7) |  | 1.00 (0.58-1.74) |  |  | 0.75 (0.39-1.42) |  |
| BMI category (kg/m^2^) |  |  |  |  | 0.62 |  |  | 0.62 |
| BMI ≥18 | 93 (95.9) | 263 (94.6) |  | 1.00 |  |  | 1.00 |  |
| BMI < 18 | 4 (4.1) | 15 (5.4) |  | 0.75 (0.24-2.33) |  |  | 0.75 (0.23-2.40) |  |
| Individual deworming (in 12 months) |  |  |  |  | 0.99 |  |  | 0.87 |
| Yes | 81 (83.5) | 232 (83.5) |  | 1.00 |  |  | 1.00 |  |
| No | 16 (16.5) | 46 (16.5) |  | 1.00 (0.54-1.86) |  |  | 0.95 (0.49-1.83) |  |
| Occupational risk |  |  |  |  | 0.028 |  |  | 0.040 |
| No | 44 (45.4) | 162 (58.3) |  | 1.00 |  |  | 1.00 |  |
| Yes | 53 (54.6) | 116 (41.7) |  | 1.68 (1.06-2.68) |  |  | 1.69 (1.02-2.78) |  |

BMI, body mass index; HIV, human immunodeficieny virus; Helminth infection risk occupation (working in the rice fields, car wash, rice harvest and fishing)

Logistic regression model was used, including the independent variables TB status, age-group, sex, HIV status, BMI, education level, employment status, number of people living in the same household, individual deworming status, occupational risk and income level in quartiles.
