# Supplementary material for: Prevalence and clinical relevance of helminth co-infections among tuberculosis patients in urban Tanzania
Source: PLoS Negl Trop Dis. 2017 Feb 8;11(2):e0005342. doi: 10.1371/journal.pntd.0005342 (PMC5319816; doi:10.1371/journal.pntd.0005342)
Supplement: S8 Table — (DOCX) [file pntd.0005342.s008.docx]

**Title: Prevalence and Clinical Relevance of Helminth Co-infections among Tuberculosis Patients in Urban Tanzania**

**S8 Table. Full table with unadjusted and adjusted odds ratios: Associations of TB disease with *Strongyloides stercolaris* and hookworm infections comparing TB patients and household contact controls without TB.**

| Characteristic |  |  | *Strongyloides stercolaris* (n=972) | | | |  | *Hookworm* infection (n=972) | | | | |
| --- | --- | --- | --- | --- | --- | --- | --- | --- | --- | --- | --- | --- |
|  | TB patient | Control |  |  |  |  |  |  |  |  |  |  |
|  | n (%) | n (%) | OR (95% CI) | p-value | aOR (95% CI) | p-value |  | OR (95% CI) | p-value |  | aOR (95% CI) | p-value |
| Helminth infection |  |  |  | 0.03 |  | 0.29 |  |  | 0.717 |  |  | 0.34 |
| No | 407 (68.2) | 278 (74.1) | 1 |  | 1.00 |  |  | 1.00 |  |  | 1.00 |  |
| Yes | 190 (31.8) | 97 (25.9) | 1.49 (1.03-2.13) |  | 1.27 (0.81-1.99) |  |  | 1.09 (0.69-1.72) |  |  | 1.31 (0.75-2.3) |  |
| Age group (years) |  |  |  | 0.081 |  | 0.25 |  |  | 0.081 |  |  | 0.25 |
| 18-24 | 107 (17.9) | 87 (23.2) | 1.00 |  | 1.00 |  |  | 1.00 |  |  | 1.00 |  |
| 25-34 | 226 (37.9) | 121 (32.3) | 1.52 (1.06-2.17) |  | 1.23 (0.77-1.95) |  |  | 1.52 (1.06-2.17) |  |  | 1.24 (0.78-1.97) |  |
| 35-44 | 169 (28.3) | 97 (25.9) | 1.42 (0.97-2.07) |  | 1.00 (0.6-1.66) |  |  | 1.42 (0.97-2.07) |  |  | 1.00 (0.60-1.67) |  |
| ≥45 | 95 (15.9) | 70 (18.7) | 1.10 (0.73-1.68) |  | 0.84 (0.48-1.47) |  |  | 1.10 (0.73-1.68) |  |  | 0.86 (0.49-1.49) |  |
| Sex |  |  |  | <0.001 |  | <0.001 |  |  | <0.001 |  |  | <0.001 |
| Female | 186 (31.2) | 201 (53.6) | 1.00 |  | 1.00 |  |  | 1.00 |  |  | 1.00 |  |
| Male | 411 (68.8) | 174 (46.4) | 2.55 (1.96-3.33) |  | 3.12 (2.14-4.57) |  |  | 2.55 (1.96-3.33) |  |  | 3.12 (2.13-4.56) |  |
| HIV status |  |  |  | <0.001 |  | <0.001 |  |  | <0.001 |  |  | <0.001 |
| Negative | 434 (72.7) | 340 (90.7) | 1.00 |  | 1.00 |  |  | 1.00 |  |  | 1.00 |  |
| Positive | 163 (27.3) | 35 (9.3) | 3.65 (2.47-5.40) |  | 6.08 (3.78-9.78) |  |  | 3.65 (2.47-5.4) |  |  | 6.16 (3.82-9.91) |  |
| Education level |  |  |  | 0.39 |  | 0.59 |  |  | 0.39 |  |  | 0.55 |
| No/primary | 500 (83.8) | 306 (81.6) | 1.00 |  | 1.00 |  |  | 1.00 |  |  | 1.00 |  |
| Secondary/University | 97 (16.2) | 69 (18.4) | 0.86 (0.61-1.21) |  | 1.13 (0.72-1.78) |  |  | 0.86 (0.61-1.21) |  |  | 1.15 (0.73-1.8) |  |
| Employment status |  |  |  | 0.16 |  | 0.59 |  |  | 0.16 |  |  | 0.68 |
| Unemployed | 204 (34.2) | 145 (38.7) | 1.00 |  | 1.00 |  |  | 1.00 |  |  | 1.00 |  |
| Employed | 393 (65.8) | 230 (61.3) | 1.22 (0.93-1.59) |  | 0.9 (0.61-1.32) |  |  | 1.22 (0.93-1.59) |  |  | 0.92 (0.63-1.35) |  |
| Smoking status |  |  |  | <0.001 |  | 0.013 |  |  | <0.001 |  |  | 0.012 |
| No | 489 (81.9) | 342 (91.2) | 1.00 |  | 1.00 |  |  | 1.00 |  |  | 1.00 |  |
| Yes | 108 (18.1) | 33 (8.8) | 2.29 (1.51-4.46) |  | 1.92 (1.15-3.2) |  |  | 2.29 (1.51-3.46) |  |  | 1.93 (1.16-3.22) |  |
| People in the household |  |  |  | 0.29 |  | 0.021 |  |  | 0.29 |  |  | 0.018 |
| ≤3 people | 442 (74.0) | 289 (77.1) | 1.00 |  | 1.00 |  |  | 1.00 |  |  | 1.00 |  |
| >3 people | 155 (26.0) | 86 (22.9) | 1.18 (0.87-1.60) |  | 1.56 (1.07-2.28) |  |  | 1.18 (0.87-1.6) |  |  | 1.58 (1.08-2.3) |  |
| Household income (USD) |  |  |  | 0.48 |  | 0.83 |  |  | 0.49 |  |  | 0.82 |
| ≤100 | 473 (79.2) | 290 (77.3) | 1.00 |  | 1.00 |  |  | 1.00 |  |  | 1.00 |  |
| >100 | 124 (20.8) | 85 (22.7) | 0.89 (0.65-1.22) |  | 1.05 (0.70-1.57) |  |  | 0.89 (0.65-1.22) |  |  | 1.05 (0.70-1.57) |  |
| BMI category (kg/m) |  |  |  | <0.001 |  | 0 |  |  | <0.001 |  |  | <0.001 |
| BMI ≥18 | 279 (46.7) | 318 (53.3) | 1.00 |  | 1.00 |  |  | 1.00 |  |  | 1.00 |  |
| BMI <18 | 356 (94.9) | 19 (5.1) | 21.36 (13.10-34.81) |  | 23.2 (13.91-38.7) |  |  | 21.36 (13.1-34.81) |  |  | 23.53 (14.10-39.25) |  |
| Occupational risk |  |  |  | 0.880 |  | 0.28 |  |  | 0.88 |  |  | 0.25 |
| No | 322 (54.2) | 199 (54.1) | 1.00 |  | 1.00 |  |  | 1.00 |  |  | 1.00 |  |
| Yes | 272 (45.8) | 169 (45.9) | 1.02 (0.79-1.32) |  | 0.83 (0.60-1.16) |  |  | 1.02 (0.79-1.32) |  |  | 0.82 (0.59-1.15) |  |
| Individual deworming (in 12 months) |  |  |  | 0.35 |  | 0.22 |  |  | 0.21 |  |  | 0.19 |
| Yes | 484 (81.1) | 313 (83.5) | 1.00 |  | 1.00 |  |  | 1.00 |  |  | 1.00 |  |
| No | 113 (18.9) | 62 (16.5) | 1.18 (0.84-1.66) |  | 0.76 (0.49-1.18) |  |  | 0.76 (0.49-1.17) |  |  | 0.75 (0.48-1.16) |  |

HIV, Human Immunodeficieny Virus; BMI, body mass index; OR, Odds ratio; 95% CI, 95% Confidence Interval

^a^ Working in rice fields, car washing, sand harvesting and fishing.

Logistic regression model adjusted for any helminth infection/*S. mansoni*, age, sex, HIV status, BMI, education level, employment status, smoking status, number of people living in the same household, individual deworming status, helminth risk occupation and income level.
