# Supplementary material for: Prevalence and clinical relevance of helminth co-infections among tuberculosis patients in urban Tanzania
Source: PLoS Negl Trop Dis. 2017 Feb 8;11(2):e0005342. doi: 10.1371/journal.pntd.0005342 (PMC5319816; doi:10.1371/journal.pntd.0005342)
Supplement: S9 Table — (DOCX) [file pntd.0005342.s009.docx]

**Title: Prevalence and Clinical Relevance of Helminth Co-infections among Tuberculosis Patients in Urban Tanzania**

**S9 Table. Additional analysis: Helminth infection and patient characteristics associated with TB among TB patients and household contact controls, using conditional logistic regression.**

| Characteristic | Any helminth infection (n=722) | | | | | |  | *Schistosoma mansoni* infection (n=455) | | | | | | |
| --- | --- | --- | --- | --- | --- | --- | --- | --- | --- | --- | --- | --- | --- | --- |
|  | TB patient | Control |  |  |  |  |  | TB patient | Control |  |  |  |  |  |
|  | n (%) | n (%) | OR (95% CI) | p-value | aOR (95% CI) | p-value |  | n (%) | n (%) | OR (95% CI) | p-value |  | aOR (95% CI) | p-value |
| Helminth infection |  |  |  | 0.398 |  | 0.198 |  |  |  |  | 0.188 |  |  | 0.008 |
| No | 247 (71) | 278 (74.3) | 1.00 |  | 1.00 |  |  | 192 (90.6) | 229 (94.2) | 1.00 |  |  | 1.00 |  |
| Yes | 101 (29) | 96 (25.7) | 1.16 (0.82-1.64) |  | 1.45 (0.82-2.55) |  |  | 20 (9.4) | 14 (5.8) | 1.69 (0.77-3.74) |  |  | 5.29 (1.55-18.1) |  |
| Age group (years) |  |  |  | 0.1 |  |  |  |  |  |  | 0.084 |  |  | 0.0391 |
| 18-24 | 62 (17.8) | 86 (23) | 1.00 |  | 1.00 |  |  | 32 (15.1) | 59 (24.3) | 1.00 | 0.834 |  | 1.00 |  |
| 25-34 | 139 (39.9) | 121 (32.4) | 1.61 (1.07-2.44) |  | 1.13 (0.58-2.19) |  |  | 84 (39.6) | 79 (32.5) | 1.6 (0.96-2.66) |  |  | 1.76 (0.77-4.03) |  |
| 35-44 | 93 (26.7) | 97 (25.9) | 1.33 (0.86-2.06) |  | 0.87 (0.42-1.82) |  |  | 65 (30.7) | 57 (23.5) | 1.64 (0.95-2.84) |  |  | 2.04 (0.74-5.61) |  |
| ≥45 | 54 (15.5) | 70 (18.7) | 1.08 (0.67-1.75) |  | 0.55 (0.25-1.2) |  |  | 31 (14.6) | 48 (19.8) | 0.92 (0.5-1.68) |  |  | 0.56 (0.2-1.51) |  |
| Sex |  |  |  | 0 |  | 0.001 |  |  |  |  | 0 |  |  | 0.007 |
| Female | 115 (33) | 200 (53.5) | 1.00 |  | 1.00 |  |  | 74 (34.9) | 133 (54.7) | 1.00 |  |  | 1.00 |  |
| Male | 233 (67) | 174 (46.5) | 2.22 (1.64-3.01) |  | 2.37 (1.41-3.98) |  |  | 138 (65.1) | 110 (45.3) | 2.03 (1.4-2.96) |  |  | 2.44 (1.27-4.67) |  |
| HIV status |  |  |  | 0 |  | 0 |  |  |  |  | 0 |  |  | 0 |
| Negative | 250 (71.8) | 339 (90.6) | 1.00 |  | 1.00 |  |  | 144 (67.9) | 221 (90.9) | 1.00 |  |  | 1.00 |  |
| Positive | 98 (28.2) | 35 (9.4) | 4.15 (2.6-6.64) |  | 9.83 (4.64-20.85) |  |  | 68 (32.1) | 22 (9.1) | 4.56 (2.55-8.16) |  |  | 9.36 (3.85-22.73) |  |
| Education level |  |  |  | 0.645 |  | 0.944 |  |  |  |  | 0.834 |  |  | 0.143 |
| No/primary | 289 (83) | 305 (81.6) | 1.00 |  | 1.00 |  |  | 173 (81.6) | 200 (82.3) | 1.00 |  |  | 1.00 |  |
| Secondary/University | 59 (17) | 69 (18.4) | 0.91 (0.62-1.35) |  | 0.98 (0.5-1.9) |  |  | 39 (18.4) | 43 (17.7) | 1.05 (0.64-1.73) |  |  | 1.92 (0.8-4.6) |  |
| Employment status |  |  |  | 0.383 |  | 0.669 |  |  |  |  | 0.212 |  |  | 0.223 |
| Unemployed | 125 (35.9) | 145 (38.8) | 1.00 |  | 1.00 |  |  | 69 (32.5) | 101 (41.6) | 1.00 |  |  | 1.00 |  |
| Employed | 223 (64.1) | 229 (61.2) | 1.61 (1.07-2.44) |  | 1.13 (0.65-1.95) |  |  | 143 (67.5) | 142 (58.4) | 1.27 (0.87-1.86) |  |  | 1.5 (0.78-2.89) |  |
| Smoking status |  |  |  | 0 |  | 0.008 |  |  |  |  | 0.008 |  |  | 0.176 |
| No | 288 (82.8) | 341 (91.2) | 1.00 |  | 1.00 |  |  | 179 (84.4) | 223 (91.8) | 1.00 |  |  | 1.00 |  |
| Yes | 60 (17.2) | 33 (8.8) | 2.28 (1.42-3.67) |  | 2.67 (1.29-5.54) |  |  | 33 (15.6) | 20 (8.2) | 2.34 (1.21-4.5) |  |  | 2.01 (0.73-5.5) |  |
| People in the household |  |  |  | 0.091 |  | 0.007 |  |  |  |  | 0.098 |  |  | 0.008 |
| ≤3 people | 255 (73.3) | 288 (77) | 1.00 |  | 1.00 |  |  | 154 (72.6) | 189 (77.8) | 1.00 |  |  | 1.00 |  |
| >3 people | 93 (26.7) | 86 (23) | 1.39 (0.95-2.05) |  | 2.46 (1.27-4.74) |  |  | 58 (27.4) | 54 (22.2) | 1.51 (0.92-2.48) |  |  | 2.8 (1.3-6.02) |  |
| Household income (USD) |  |  |  | 0.85 |  | 0.714 |  |  |  |  | 0.44 |  |  | 0.196 |
| ≤100 | 268 (77) | 289 (77.3) | 1.00 |  | 1.00 |  |  | 163 (76.9) | 183 (75.3) | 1.00 |  |  | 1.00 |  |
| >100 | 80 (23) | 85 (22.7) | 1.04 (0.72-1.5) |  | 1.12 (0.61-2.04) |  |  | 49 (23.1) | 60 (24.7) | 0.84 (0.53-1.32) |  |  | 0.6 (0.28-1.3) |  |
| BMI category (kg/m) |  |  |  | 0 |  | 0 |  |  |  |  | 0 |  |  | 0 |
| BMI ≥18 | 167 (48) | 355 (94.9) | 1.00 |  | 1.00 |  |  | 110 (51.9) | 228 (93.8) | 1.00 |  |  | 1.00 |  |
| BMI <18 | 181 (52) | 19 (5.1) | 15.5 (8.63-27.85) |  | 21.01 (10.33-42.71) |  |  | 102 (48.1) | 15 (6.2) | 11.48 (5.79-22.77) |  |  | 19.98 (7.89-50.57) |  |
| Occupational risk |  |  |  | 0.321 |  | 0.015 |  |  |  |  | 0.807 |  |  | 0.061 |
| No | 204 (58.6) | 206 (55.1) | 1.00 |  | 1.00 |  |  | 121 (57.1) | 139 (57.2) | 1.00 |  |  | 1.00 |  |
| Yes | 144 (41.4) | 168 (44.9) | 0.85 (0.62-1.17) |  | 0.54 (0.33-0.89) |  |  | 91 (42.9) | 104 (42.8) | 0.95 (0.63-1.43) |  |  | 0.53 (0.27-1.03) |  |
| Individual deworming (in 12 months) |  |  |  | 0.552 |  | 0.18 |  |  |  |  | 0.298 |  |  | 0.278 |
| Yes | 284 (81.6) | 312 (83.4) | 1.00 |  | 1.00 |  |  | 172 (81.1) | 206 (84.8) | 1.00 |  |  | 1.00 |  |
| No | 64 (18.4) | 62 (16.6) | 1.13 (0.76-1.66) |  | 0.66 (0.36-1.21) |  |  | 40 (18.9) | 37 (15.2) | 1.31 (0.79-2.19) |  |  | 0.63 (0.27-1.45) |  |

HIV, Human Immunodeficieny Virus; BMI, body mass index; OR, Odds ratio; 95% CI, 95% Confidence Interval

^a^ For comparison with *Schistosoma mansoni*, we excluded 232 participants with any helminth infections other than *Schistosoma mansoni*.^b^ Working in rice fields, car washing, sand harvesting and fishing

Conditional logistic regression model taking into account case-control matching, and adjusted for any helminth infection/*S. mansoni*, age, sex, HIV status, BMI, education level, employment status, smoking status, number of people living in the same household, individual deworming status, helminth risk occupation and household income level.

Excluded TB patients due to unpaired to controls: 250 cases for any helminth infection, and 285 cases for *S. mansoni* infection.
