# Supplementary material for: Prevalence and clinical relevance of helminth co-infections among tuberculosis patients in urban Tanzania
Source: PLoS Negl Trop Dis. 2017 Feb 8;11(2):e0005342. doi: 10.1371/journal.pntd.0005342 (PMC5319816; doi:10.1371/journal.pntd.0005342)
Supplement: S10 Table — (DOCX) [file pntd.0005342.s010.docx]

**Title: Prevalence and Clinical Relevance of Helminth Co-infections among Tuberculosis Patients in Urban Tanzania**

**S10 Table. Radiological findings of chest X-rays in TB patients at the time of TB diagnosis, stratified by helminth infection status.**

| Radiological findings | Total | TB and helminths | TB only |  |
| --- | --- | --- | --- | --- |
|  | **(n=335)** | **(n=93**) | **(n=242)** | p-value^a^ |
| Infiltrations | 276 (82.4) | 81 (87.1) | 195 (80.6) | 0.16 |
| Unilateral | 196 (71.0) | 55 (67.9) | 141 (72.3) |  |
| Bilateral | 80 (29.0) | 26 (32.1) | 54 (27.7) |  |
| Cavitations | 146 (43.6) | 45 (48.4) | 101 (41.7) | 0.27 |
| Unilateral | 132 (90.4) | 38 (84.4) | 94 (93.1) |  |
| Bilateral | 14 (9.6) | 7 (15.6) | 7 (6.9) |  |
| Micronodules (< 7mm) | 26 (7.8) | 9 (9.7) | 17 (7.0) | 0.42 |
| Unilateral | 11 (42.3) | 5 (55.6) | 6 (35.3) |  |
| Bilateral | 15 (57.7) | 4 (44.4) | 11 (64.7) |  |
| Macronodules (≥7mm) | 7 (2.1) | 1 (1.1) | 6 (2.5) | 0.42 |
| Unilateral | 5 (71.4) | 0 (0) | 5 (83.3) |  |
| Bilateral | 2 (28.6) | 1 (100) | 1 (16.7) |  |
| Pleural effusion | 52 (15.5) | 11 (11.8) | 41 (16.9) | 0.25 |
| Unilateral | 46 (88.5) | 9 (81.8) | 37 (90.2) |  |
| Bilateral | 6 (11.5) | 2 (18.2) | 4 (9.8) |  |
| Pulmonary edema | 18 (5.4) | 2 (2.2) | 16 (6.6) | 0.11 |
| Unilateral | 4 (22.2) | 1 (50.0) | 3 (18.8) |  |
| Bilateral | 14 (77.8) | 1 (50.0) | 13 (81.3) |  |
| Lymph node enlargements (intrathoracic) | 38 (11.3) | 8 (8.6) | 30 (12.4) | 0.33 |
| Radiologist severity grading ^b^ |  |  |  | 0.76 |
| Mild/moderate | 246 (73.4) | 67 (72.0) | 179 (74.0) |  |
| Severe | 88 (26.3) | 26 (28.0) | 62 (25.6) |  |

^a^ Pearson chi-squared test; ^b^ Radiologist grading of the chest-ray severity
