# Supplementary material for: Prevalence and clinical relevance of helminth co-infections among tuberculosis patients in urban Tanzania
Source: PLoS Negl Trop Dis. 2017 Feb 8;11(2):e0005342. doi: 10.1371/journal.pntd.0005342 (PMC5319816; doi:10.1371/journal.pntd.0005342)
Supplement: S11 Table — (DOCX) [file pntd.0005342.s011.docx]

**Title: Prevalence and Clinical Relevance of Helminth Co-infections among Tuberculosis Patients in Urban Tanzania**

**11 Table. Association of helminth infection with poor recovery of BMI, poor gain of absolute weight, and percentage body fat in TB patients, between recruitment and after six months of completed TB treatment.**

| Helminth infection | Poor BMI gain | |  | Poor weight gain | |  | Poor gain in percentage body fat | |
| --- | --- | --- | --- | --- | --- | --- | --- | --- |
|  | aOR (95% CI) | p-value |  | aOR (95% CI) | p-value |  | aOR (95% CI) | p-value |
| Any helminth |  | 0.23 |  |  | 0.63 |  |  | 0.78 |
| No | 1.00 |  |  | 1.00 |  |  | 1.00 |  |
| Yes | 0.74 (0.46-1.21) |  |  | 0.89 (0.55-1.45) |  |  | 0.92 (0.55-1.56) |  |
| *Strongyloides stercolaris* |  | 0.49 |  |  | 0.11 |  |  | 0.34 |
| No | 1.00 |  |  | 1.00 |  |  | 1.00 |  |
| Yes | 0.81 (0.44-1.47) |  |  | 0.62 (0.34-1.12) |  |  | 0.73 (0.39-1.38) |  |
| *Schistosoma mansoni* |  | 0.35 |  |  | 0.77 |  |  | 0.62 |
| No | 1.00 |  |  | 1.00 |  |  | 1.00 |  |
| Yes | 0.68 (0.29-1.58) |  |  | 0.88 (0.38-2.03) |  |  | 0.80 (0.33-1.92) |  |
| Hookworm |  | 0.44 |  |  | 0.79 |  |  | 0.91 |
| No | 1.00 |  |  | 1.00 |  |  | 1.00 |  |
| Yes | 0.72 (0.30-1.68) |  |  | 0.89 (0.37-2.12) |  |  | 0.95 (0.39-2.32) |  |
| Multiple infections |  | 0.27 |  |  | 0.44 |  |  | 0.96 |
| None | 1.00 |  |  | 1.00 |  |  | 1.00 |  |
| Mono | 0.67 (0.40-1.13) |  |  | 0.99 (0.59-1.68) |  |  | 0.92 (0.52-1.62) |  |
| Double or more | 1.17 (0.44-3.11) |  |  | 0.54 (0.21-1.41) |  |  | 0.93 (0.35-2.53) |  |

Poor gain was defined defines as BMI change <2.6 kg/m^2^, poor weight change, as weight gain <7 kg; poor body fat gain as percentage body fat <0.0%

Logistic regression model adjusted for: age, sex, HIV status, number of people in the household and income level quartiles.

Patients with any other helminth infection were excluded: 43 TB patients for *S. stercolaris*; 74 TB patients for *S. mansoni*; 75 TB patients for hookworm.
