# Supplementary material for: Prevalence and clinical relevance of helminth co-infections among tuberculosis patients in urban Tanzania
Source: PLoS Negl Trop Dis. 2017 Feb 8;11(2):e0005342. doi: 10.1371/journal.pntd.0005342 (PMC5319816; doi:10.1371/journal.pntd.0005342)
Supplement: S1 Fig — (A) The prevalence of helminth infection summarized at the ward level. (B) The helminth species distribution at the study area. Other helminth infections include: Ascaris lumbricoides, Enterobius vermicularis, Trichuris trichiura, and Hymenolepis diminuta. (DOCX) [file pntd.0005342.s012.docx]

**Title: Prevalence and Clinical Relevance of Helminth Co-infections among Tuberculosis Patients in Urban Tanzania**

| **A** | 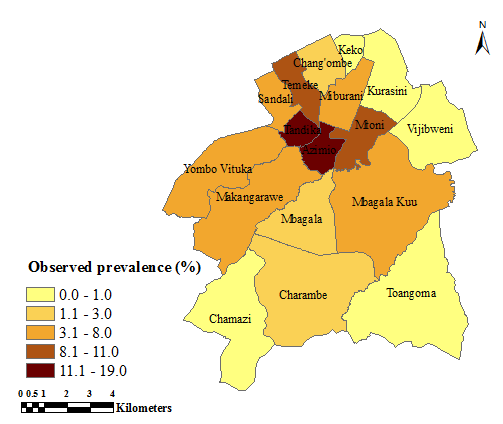 |
| --- | --- |
| **B** | 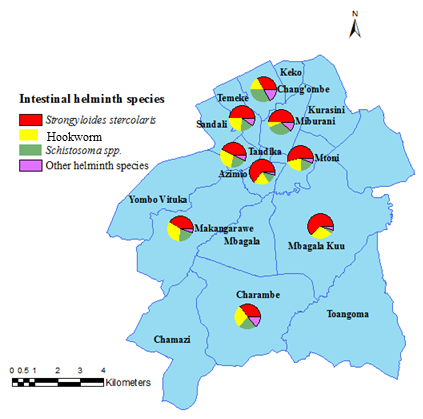  **S1 Figure: Geographical distribution of helminth infections in the study area.** (A) The prevalence of helminth infection summarized at the ward level. (B) The helminth species distribution at the study area. Other helminth infections include: *Ascaris lumbricoides*, *Enterobius vermicularis*, *Trichuris trichiura* and *Hymenolepis dimunita.* |
